# Evaluating the Effectiveness of InsightApp for Anxiety, Valued Action, and Psychological Resilience: Longitudinal Randomized Controlled Trial

**DOI:** 10.2196/57201

**Published:** 2025-02-04

**Authors:** Victoria Amo, Falk Lieder

**Affiliations:** 1 Max Planck Institute for Intelligent Systems Tübingen Germany; 2 Department of Psychology UCLA Los Angeles, CA United States

**Keywords:** ecological momentary intervention, serious game, mindfulness-based intervention, acceptance and commitment therapy, cognitive behavioral therapy, metacognition, stress, anxiety, mobile phone

## Abstract

**Background:**

Anxiety disorders are among the most prevalent mental disorders, and stress plays a significant role in their development. Ecological momentary interventions (EMIs) hold great potential to help people manage stress and anxiety by training emotion regulation and coping skills in real-life settings. InsightApp is a gamified EMI and research tool that incorporates elements from evidence-based therapeutic approaches. It is designed to strengthen people’s metacognitive skills for coping with challenging real-life situations and embracing anxiety and other emotions.

**Objective:**

This randomized controlled trial aims to examine the effectiveness of InsightApp in (1) improving individuals’ metacognitive strategies for coping with stress and anxiety and (2) promoting value-congruent action. It also evaluates how long these effects are retained. This experiment advances our understanding of the role of metacognition in emotional and behavioral reactivity to stress.

**Methods:**

We conducted a randomized controlled trial with 228 participants (completion rate: n=197, 86.4%; mean age 38, SD 11.50 years; age range 20-80 years; female: n=101, 52.6%; and White: n=175, 91.1%), who were randomly assigned to either the treatment or the active placebo control group. During the 1-week intervention phase, the treatment group engaged with InsightApp, while participants in the control group interacted with a placebo version of the app that delivered executive function training. We assessed the differences between the 2 groups in posttest and follow-up assessments of mental health and well-being while controlling for preexisting differences. Moreover, we used a multilevel model to analyze the longitudinal data, focusing on the within-participant causal effects of the intervention on emotional and behavioral reactivity to daily stressors. Specifically, we measured daily anxiety, struggle with anxiety, and value-congruent action.

**Results:**

The intervention delivered by InsightApp yielded mixed results. On one hand, we found no significant posttest scores on mental health and well-being measures directly after the intervention or 7 days later (all *P*>.22). In contrast, when confronted with real-life stress, the treatment group experienced a 15% lower increase in anxiety (1-tailed *t* test, *t*_197_=–2.4; *P*=.009) and a 12% lower increase in the struggle with anxiety (*t*_197_=–1.87; *P*=.031) than the control group. Furthermore, individuals in the treatment group demonstrated a 7% higher tendency to align their actions with their values compared to the control group (*t*_197_=3.23; *P*=.002). After the intervention period, InsightApp’s positive effects on the struggle with anxiety in reaction to stress were sustained, and increased to an 18% lower reactivity to stress (*t*_197_=–2.84; *P*=.002).

**Conclusions:**

As our study yielded mixed results, further studies are needed to obtain an accurate and reliable understanding of the effectiveness of InsightApp. Overall, our findings tentatively suggest that guiding people to apply adaptive metacognitive strategies for coping with real-life stress daily with a gamified EMI is a promising approach that deserves further evaluation.

**Trial Registration:**

OSF Registries osf.io/k3b5d; https://osf.io/k3b5d

## Introduction

### Background

According to the World Health Organization, anxiety disorders currently affect approximately 3.6% of the world’s population, which translates to about 264 million individuals worldwide, making them one of the most common mental health conditions globally [1]. Moreover, in the United States, approximately 31.1% of adults experience some form of anxiety disorder during their lives [2]. These numbers do not include the distress experienced by individuals dealing with subclinical levels of anxiety daily. Anxiety often arises when people feel threatened or face stressful situations. Prolonged or intense stress can disrupt the body’s stress response system, leading to heightened anxiety levels [3,4]. By regulating the stress response system, individuals can learn to modulate their reactivity to stressors, reduce anxiety symptoms, and enhance overall well-being [5-7].

Ecological momentary interventions (EMIs) are digital interventions delivered in real time in real-world settings to help individuals integrate specific strategies into their everyday life [8]. The widespread use of smartphones and other mobile devices makes EMIs easily accessible and provides a broad reach. Therefore, EMIs offer a scalable, cost-effective, and flexible platform for delivering mental health interventions, including psychotherapy [9,10]. In particular, EMIs have great potential for helping individuals cope with stress in real time, because they can provide immediate support and resources to address stressors as they arise throughout the day. Consequently, EMIs present a promising avenue for enhancing therapeutic outcomes and promoting mental well-being by giving the general public access to important tools and strategies for psychological resilience and well-being.

One approach that is consistently emphasized in various psychotherapies, including metacognitive therapy [11], cognitive behavioral therapy (CBT) [12], acceptance and commitment therapy (ACT) [13], mindfulness-based stress reduction [14], and various mindfulness-based interventions [15], is the development of metacognitive skills. Metacognitive skills allow individuals to effectively navigate and regulate their cognitive and emotional processes, leading to improved coping strategies and overall well-being [6,16-18]. Therefore, combining the benefits of metacognitive skill training with the accessibility and effectiveness of EMIs is a promising avenue to helping individuals actively cultivate self-awareness, regulate their cognitive processes, and integrate specific coping strategies into their daily lives.

InsightApp is a gamified EMI and research tool designed to strengthen people’s metacognitive skills for coping with challenging real-life situations and embracing strong emotions [19]. The main strategies the app integrates are the *activating event, belief system, consequences, disputation of beliefs, and effective new beliefs* *(ABCDE) method* for cognitive restructuring from CBT; reflections on values and committed action from ACT; and emotion regulation strategies from ACT, CBT, and mindfulness-based interventions.

In addition to the benefits of helping individuals improve their mental well-being, EMIs facilitate running longitudinal studies with the help of ecological momentary assessment. This approach allows researchers to collect real-time data on the effectiveness of interventions over extended periods [20]. A longitudinal approach addresses methodological shortcomings, such as retrospective recall bias, and enables examination of both between-participant (personality level) differences and within-participant variations [21]. By incorporating longitudinal designs and daily assessments in real-life settings, researchers can gain valuable insights into the dynamic nature of stress and coping, contributing to the development of more effective and personalized interventions.

### Objectives

This experiment aimed to conduct a longitudinal randomized controlled trial to evaluate the effectiveness of InsightApp in enhancing individuals’ mental health and well-being as well as addressing their daily anxiety levels and their capacity to engage in valued actions when confronted with challenging situations. Our experiment evaluates the impact of the intervention on mental health and well-being before and after the intervention. Furthermore, through a longitudinal experimental design with a robust placebo control group, we gain insights into how InsightApp affects participants’ anxiety, their ability to cope with stress, and their engagement in valued actions in everyday life.

## Methods

### InsightApp

#### Overview

InsightApp was designed and programmed by VA as part of her PhD project at the Max Planck Institute for Intelligent Systems under the supervision of FL. The app was developed for academic purposes without any affiliation to commercial interests. It serves a dual purpose: studying metacognitive mechanisms behind belief, behavior, and emotion regulation and enhancing individuals’ skills in these areas. The development of InsightApp involved a series of iterative processes, incorporating feedback from formative evaluations and usability testing. Detailed information about its formative stages and the impact of these evaluations on the app’s design can be found in a previous publication [19].

The primary components of the intervention delivered by InsightApp are the reflection modules and the metacognitive coaches, which are explained in detail subsequently.

#### Reflection Modules

InsightApp comprises 3 reflection modules: reactivity, values, and choice point. The reactivity module focuses on the ABCDE method (activating events and consequences) for cognitive restructuring. It guides participants to reflect on a challenging situation and to identify their typical reactions (Table S1 in Multimedia Appendix 1). The values module, based on ACT, guides users to identify the personal values that they would like to express in their situation. The app assists users in discovering a meaningful course of action by offering suggestions aligned with their chosen values. The choice point module draws from ACT’s concept of choice points (Table S3 in Multimedia Appendix 1). It presents individuals with divergent paths, where one leads to growth and valued action, while the other leads to old habits and reactive behavior.

#### Metacognitive Coaches

InsightApp trains 2 types of metacognitive skills with the help of 2 coaches: the meta-reasoning coach and the meta-awareness coach. The meta-reasoning coach trains participants’ capacity to think critically about the content of their thoughts as subjective interpretations of their reality. The meta-awareness coach trains participants’ capacity to notice, accept, and kindly embrace their thoughts and emotions as mental events as they occur in real time.

**Figure 1:**
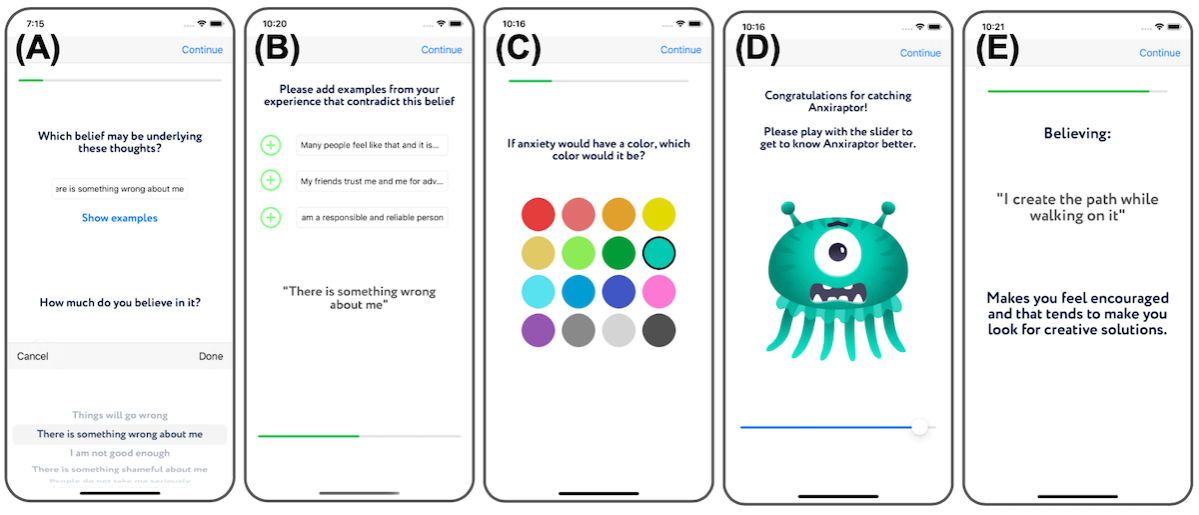
Screenshots of the meta-reasoning coach. The figure shows example screens illustrating how the app guides the user to (A) reflect on the content of their thoughts when experiencing a strong negative emotion, (B) find evidence that contradicts the maladaptive belief, (C and D) personalize a "little monster" avatar to represent the pattern, and (E) ponder the influence of an alternative helpful adaptive belief on their emotions and behavioral tendencies.

**Figure 2:**
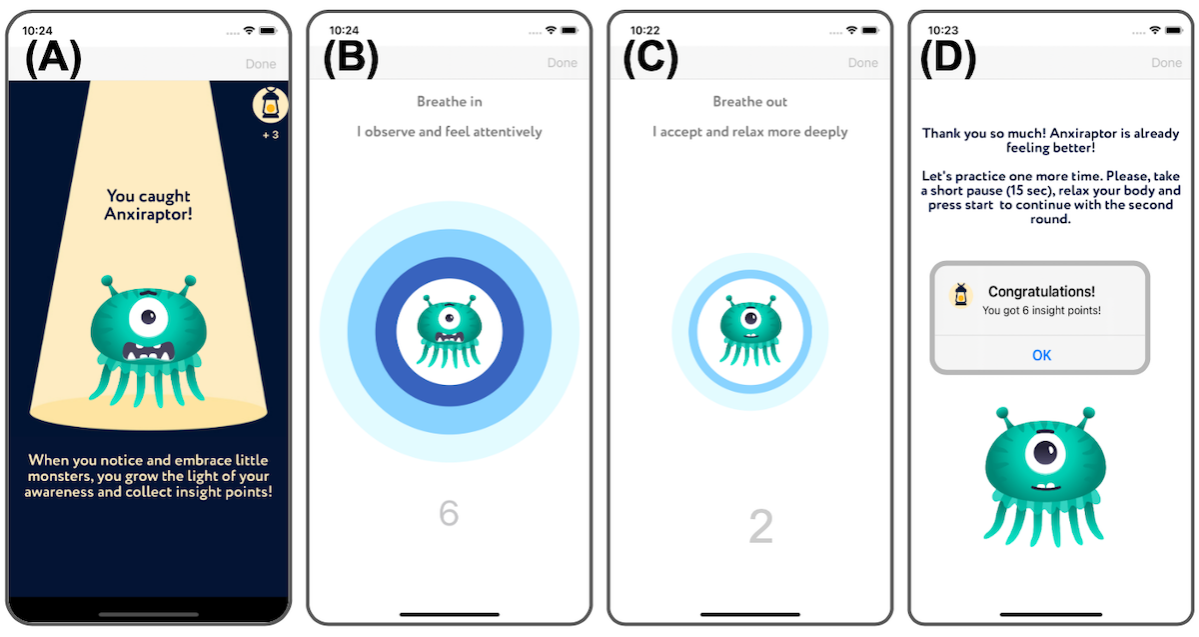
Screenshots of the meta-awareness coach. The figure shows example screens illustrating how the app (A) introduces users to insight point rewards and guides the user to (B) inhale while observing how the emotion feels in the body, (C) exhale while accepting those sensations and relaxing, and (D) take a short break between breathing sessions where they are awarded with insight point rewards.

The meta-reasoning coach integrates steps B, C, D, and E of the ABCDE method for cognitive restructuring. It helps users identify anxiety-inducing thoughts (Figure 1A) and their consequences (Figure 1B) and challenge their negative thoughts (Figure 1C). Furthermore, the coach prompts the user to customize a “little monster” avatar to represent the combination of anxious feelings, thoughts, and actions identified in the previous steps (Figures 1D and 1E). It also assists them in finding an adaptive way to think about their situation and understanding how the adaptive belief influences the way they feel and act. Table S4 in Multimedia Appendix 1 details the coach’s questions and answer types. The meta-awareness coach leads users through a breathing meditation on acceptance. In the initial session, users are introduced to insight point rewards (Figure 2A), which encourage a positive attitude toward challenges and frame them as opportunities for practicing emotion regulation skills (Figure 2A). The meditation guides users to observe anxious body sensations, symbolized by the avatar, during inhalation, with expanding rings (Figure 2B) and to accept these sensations and relax during exhalation, accompanied by contracting rings (Figure 2C). At the end of every practice session, users receive a congratulatory message and are awarded insight point rewards (Figure 2D). The meditation consists of 2 sessions, each comprising 6 breaths.

The app, as an EMI, prompts people to integrate the training of metacognitive skills into their daily lives. The app encourages participants to stay aware of their emotions and thoughts throughout the day (metacognitive awareness practice) and to “catch” the little monster avatar whenever they recognize themselves experiencing or acting in anxious ways they have previously identified (decentering or cognitive defusion practice). Participants are presented with a choice point immediately after completing the cognitive defusion practice. At this point, the app prompts participants to click on the course of action they choose to take next. Furthermore, the app sends 2 random notifications per day to remind participants to stay aware of their emotions and to use the catch function when they notice themselves feeling anxious or acting in the reactive ways they have previously identified. These notifications serve as prompts to practice emotional awareness throughout the day. Thereby, the app prompts users to integrate metacognitive skill training into their daily routines through real-time notifications and personalized content of the intervention that adapts to the users’ specific situations, emotions, and tendencies to action. Table S5 in Multimedia Appendix 1 provides the details of the EMI functionalities of the app.

The app incorporates gamification by guiding participants to create an avatar that represents their emotions, embedding metacognitive skill training within a narrative framework, and awarding insight points for catching the little monster associated with identified anxious thoughts or behaviors. This approach uses game elements, such as rewards and interactive storytelling, to transform the training process into a game-like experience, fostering engagement and motivation through personalized challenges and immediate feedback.

### Ethical Considerations

The experiment was conducted under protocol number 510/2020BO approved by the Independent Ethics Commission at the Medical Faculty of the University of Tübingen. The Max Planck Society Data Protection Office has granted authorization for the data protection and privacy policy protocol. This authorization confirms that the project proposal adheres to the internal data protection requirements of the Max Planck Society as well as the General Data Protection Regulation (EU 2016/679) and the German Federal Data Protection Act. The data protection and privacy policy agreements are incorporated in both the ethics protocol and the consent form.

To enroll in the experiment, participants provided informed consent to (1) their voluntary participation in the experiment and (2) the sharing of their nonidentifiable data for research purposes. By giving their consent, participants agreed to adhere to the requirements for participation, including aspects such as payments, bonuses, time commitment, privacy policy, data protection, data collection, data use, and the withdrawal process. To ensure privacy, all data collected during the study are stored locally on participants' devices and synchronized securely with Firebase (Google) cloud services, certified under major privacy and security standards. Only the participant and experimenters have access to these data. Participants can withdraw their participation at any time without penalty, with compensation provided pro rata to the time engaged in the study.

The experiment’s payment was split into 2 parts. The initial payment was given to participants upon downloading the app and finishing the 30-minute onboarding. The payment was adjusted to maintain an average wage of GBP £6 (US $7.58) per hour. After completing the experiment, participants were compensated for daily participation, offboarding tasks, and extra bonuses. Through active participation and successful completion of the experiment, participants could earn up to GBP £9.80 (US $10.59) per hour. Excluded participants were compensated for the work completed before their exclusion, using a rate of GBP £6 (US $7.58) per hour. The estimated average experiment time was 154 (SD 8.85) minutes for participants in the control group and 161 (SD 8.04) minutes for those in the treatment group.

### Participants

The sample for this experiment comprised 228 participants, of whom 197 (86.4%) completed the experiment. Participants were recruited from a pool of approximately 39,000 English-speaking adults with iPhones. We used Prolific (Prolific Academic Ltd), a web-based platform used by >25,000 researchers, to recruit and compensate these individuals. Prolific offers access to a diverse pool of >130,000 potential participants, enabling efficient web-based research engagement.

In our study, participants were enrolled at 2 separate points. Initially, 228 participants were enrolled at the start of the study. To compensate for the participants who were excluded and those who dropped out before the experimental conditions diverged on day 3, as preregistered, we conducted a second round of recruitment to maintain the required sample size. Specifically, of the 228 participants, 22 (9.6%) were excluded for failing attention checks, 13 (5.7%) for not starting the experiment, and 2 (0.9%) due to technical issues. These 37 (16.2%) participants were replaced by new enrollees.

During the experiment, 30 (13.2%) of the 228 participants were lost to follow-up due to not meeting the participation criteria (n=22, 9.6%) or technical problems (n=8, 3.5%). In addition, 1 participant (0.4%) was excluded from the data analysis for failing midexperiment attention checks. Multimedia Appendix 2 includes a detailed description of the inclusion and exclusion criteria, along with the recruitment and data collection timeline (Table S1 in Multimedia Appendix 2).

The final sample of 197 participants comprised 97 participants (49.2%) in the experimental condition and 100 participants (50.8%) in the control condition. Most (192/197, 97.5%) of these participants shared demographic information (mean age 38, SD 11.50 years; range 20-80 years; female: n=103, 53.6%). The sample included participants who identified as Asian (7/192, 3.6%), Black (3/192, 1.6%), mixed race (5/192, 2.6%), White (175/192, 91.1%), or other (2/192, 1%). More detailed demographic characteristics, including descriptive statistics by group, are provided in Multimedia Appendix 3.

### Experimental Design

#### Overview

The experiment design and data analysis plan were preregistered to ensure transparency and minimize biases in the analysis and interpretation of the results. Details of the study methodology and reporting adhere to the CONSORT (Consolidated Standards of Reporting Trials) checklist, provided as Multimedia Appendix 4. We conducted a placebo-controlled randomized trial to assess the efficacy of the longitudinal intervention delivered by InsightApp. The experiment encompassed 2 groups: a placebo control group and a treatment group. The experiment used 2 distinct experimental designs: a pre-post follow-up design and a longitudinal experience sampling design. Each experimental design encompasses different sets of outcome measures. The pre-post follow-up design administered self-report measures of mental health and well-being at 3 time points: before the intervention, immediately after the intervention, and after the postintervention phase. The longitudinal experience sampling methods involved multiple daily assessments of anxiety throughout the 18-day experimental period, which included a 4-day preintervention phase, a 7-day intervention phase, and a 7-day postintervention phase.

#### Experiment Timeline

The experiment timeline encompassed 6 distinct phases (Table 1), including general onboarding, preintervention, intervention, midintervention assessments, postintervention, and offboarding.

**Table 1:** Phases of the experiment timeline.

	Onboarding phase	Preintervention phase (4 days)	Intervention phase (7 days)	Midintervention assessments	Postintervention phase (7 days)	Offboarding phase
Treatment group	Reflection module	Evening survey	Morning practice Cognitive defusion practice Evening survey	Psychological scales Motivation survey	Evening survey	Psychological scales Exit survey
Placebo control group	Reflection module	Evening survey	Evening survey	Psychological scales	Evening survey	Psychological scales Exit survey

During the onboarding phase (phase 1), all participants completed the reflection module. During the 4-day preintervention phase (phase 2), participants in both conditions used a basic version of the app that only allowed them to complete the daily evening report between 7:00 PM and 11:59 PM. During the 7-day intervention phase (phase 3), participants continued to complete the evening report. In addition, participants in the experimental condition were introduced to the metacognitive coaches, while participants in the control condition were introduced to execute function tasks described in the Experimental Design section. During this intervention phase, each group completed a practice session every morning between 5 AM and 11:59 AM. In this session, the treatment group interacted with the meta-awareness coach to practice embracing anxiety. In contrast, the placebo control group used the app to keep the training of executive functions. Besides the morning practice, the treatment group was asked to use InsightApp to embrace their emotions throughout the day. Directly after the intervention period (phase 4), all participants retook the psychological surveys. During the 7-day postintervention phase (phase 5), all participants were requested to solely complete the evening report daily. In the offboarding phase (phase 6), participants retook the psychological surveys, filled out an exit survey, and received their payment. A detailed breakdown of the main tasks and their timing for each round of participants is provided in Table S1 in Multimedia Appendix 2.

#### Design of the Placebo Control Condition

**Figure 3:**
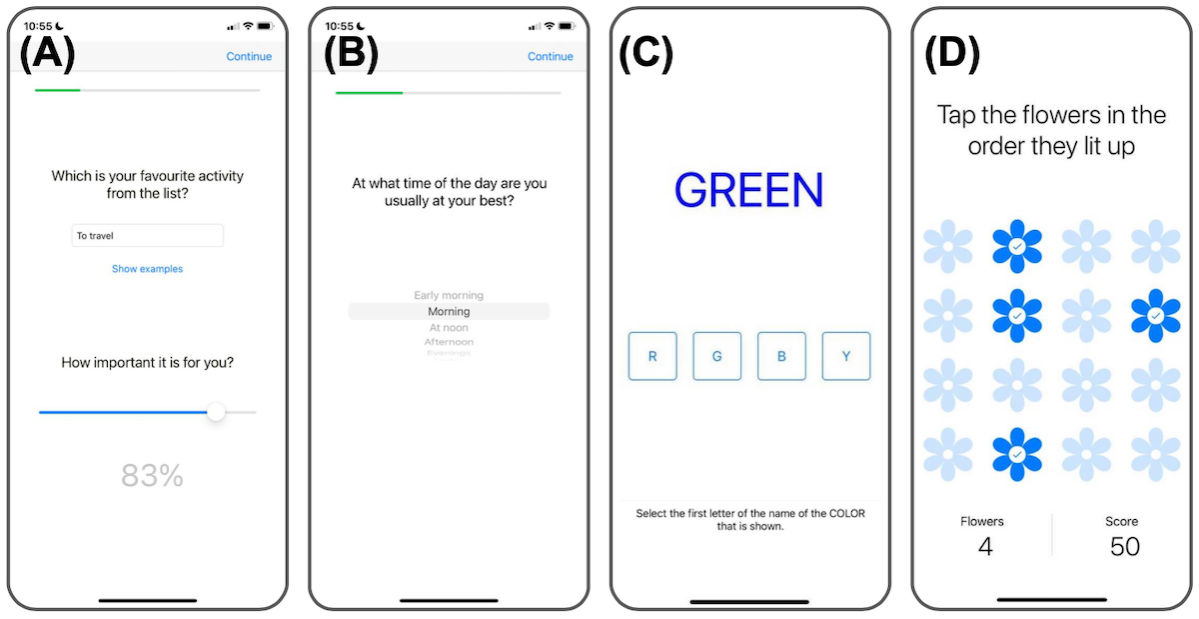
Screenshots from the control condition’s tasks. Panels A and B show example screens illustrating how the app guides the user to answer unrelated questions regarding their preferences, panel C shows the Stroop task, and panel D shows the spatial memory task.

Previous research highlights the importance and challenges of creating robust control conditions to account for improvements unrelated to digital interventions [22,23]. Accordingly, we designed the control condition to address improvements influenced by participants’ implicit goal to improve their emotional states, which can trigger emotion regulation by itself (emotion goals [24]), participants’ potential motivation to align with the experiment’s objectives (demand characteristics [25]), and participants’ expectations for the digital intervention to be effective (digital placebo effect [23]). In this active placebo control condition, the active ingredients of the intervention were replaced by executive function training exercises and self-report questionnaires. Specifically, the placebo condition differed from the treatment only in the specific components under the study. The treatment group worked with the meta-awareness and meta-reasoning coach, while the control group answered questions about psychological traits, preferences, and attentional skills (refer to Figures 3A and 3B). A complete list of control group questions has been provided in Table S1 in Multimedia Appendix 5. In addition, the placebo control group replaced the meta-awareness coach with a Stroop task (Figure 3C) and a spatial memory task (Figure 3D) sourced from the ResearchKit (Apple Inc) library [26]. These tasks enhance cognitive control and attentional skills linked to improved well-being [27-29]. Multimedia Appendix 5 provides a detailed description of the active placebo control condition and how it addresses emotion goals, demand characteristics, and the digital placebo effect.

### Procedure

Participants were first directed to a web-based form, which provided instructions for downloading InsightAppExperiment from the App Store and starting the experiment. Participants were randomly assigned to the control or treatment group through an automated process within the app. Participants were blinded to their group allocation. Further details on the randomization and blinding process are provided in Multimedia Appendix 6. After logging into the app, participants provided informed consent, confirming their agreement to participate in the experiment and share their data. The experiment started with a brief 1-minute introductory video that explained the onboarding process, followed by a short survey about the participants’ sex, age, and nationality. To proceed, all participants completed the reactivity and values reflection modules. Participants then set reminder preferences and received payment instructions. Finally, they accessed the app’s main screen to initiate the preintervention phase. They then accessed the app’s main screen to begin the experimental timeline explained earlier.

During the study, participant engagement was actively monitored. Participants received daily reminders within the app to complete their morning routines and fill out their evening reports. In addition, we sent daily messages via Prolific to remind them to participate in the study. If participants did not complete an evening report, they were allowed to do so the following morning.

### Outcome Measures

#### Pre- and Posttest Measures: Questionnaire Measures of Mental Health and Well-Being

##### Overview

We evaluated participants’ mental well-being and mental health at 3 different time points: during the onboarding phase (preintervention phase), immediately after the intervention (postintervention phase), and directly after the postintervention (follow-up phase). All measures were administered as web-based surveys within the app. This assessment was conducted using the psychological surveys mentioned subsequently.

##### Mental Well-Being

#### Thriving

The Brief Inventory of Thriving Scale [30] is a 10-item, 5-point scale (1=strongly disagree and 5=strongly agree) that measures various aspects of psychological well-being. It assesses 10 facets of positive functioning, representing 7 dimensions of psychological well-being.

#### Self-Compassion

The Self-compassion Scale (Short form) [31] is a 12-item, 5-point scale (1=never disagree and 5=always) that assesses individuals’ ability to embrace their feelings of emotional pain with a sense of warmth, connection, and concern.

#### Psychological Flexibility

The Acceptance and Action Questionnaire [32] is a 7-item, 7-point scale (1=never true and 7=always true) that assesses individuals’ ability to fully experience the present moment, thoughts, and feelings without resistance and to adapt their behavior in pursuit of goals and values as needed.

##### Mental Health

#### Anxiety Sensitivity

The Anxiety Sensitivity Index [33] is a 16-item, 5-point scale (1=very little and 7=very much) that measures individuals’ fear of sensations of anxiety.

#### Anxiety Symptoms

The Hamilton Anxiety Rating Scale (HAM-A) [34] is a 14-item, 5-point scale (1=not present and 5=very severe) used to assess the severity of anxiety symptoms, both psychological and physical.

#### Cognitive Fusion With Anxiety

The Believability of Anxious Feelings and Thoughts Questionnaire [35] is a 16-item, 7-point scale (1=not at all believable and 7=completely believable) used to measure the extent to which individuals cognitively fuse with their anxious thoughts and feelings.

#### Neuroticism

The Brief Version of the Big Five Personality Inventory [36] is a 10-item, 5-point scale (1=disagree strongly and 5=agree strongly) that assesses various personality traits, including neuroticism. In this experiment, we only administered the 2-item subscale on neuroticism.

##### Longitudinal Measures: Outcome Measures Administered in the Evening Report

Every evening, between 7:00 PM and 11:59 PM, participants reported how intense their anxiety felt during the day, the degree to which they struggled with anxiety, the degree to which they enacted the predefined valued action, the perceived severity of the challenging situation encountered during the day (stressor), and the presence of any unrelated circumstances causing additional anxiety (unrelated stressor). The outcome measures collected through these reports are summarized in Table 2.

**Table 2:** Single-item outcome measures administered during the evening report^a^.

Outcome variable	Question	Scale
Anxiety intensity (%)	How intense did anxiety feel today?	0-100
Struggle with anxiety (%)	How strong did the struggle with anxiety feel today?	0-100
Intention enactment (%)	To which extent did enact a valued action today?	0-100
Stressor	On a scale from 1 to 10, how severe or problematic was the situation today?	0-10
Unrelated stressor	On a scale from 1 to 10, did you have another situation causing you anxiety today? How severe or problematic was it?	0-10

^a^We used the measure of single-item sliding scales proposed by Amo et al [19] for momentary and daily levels of anxiety, struggle with anxiety, unwanted action, valued action, and the endorsement of the maladaptive and adaptive beliefs.

In this study, we operationalized resilience as participants’ capacity to regulate emotional intensity and struggle in response to increasing stress. Specifically, we measured each group’s average level of resilience by how little their anxiety intensity and struggle with anxiety increased with within-person variation in stress. This allowed us to measure the intervention’s effect on resilience by the coefficient of the interaction effect between the experimental condition and within-person variation in stress. We evaluated whether participants in the intervention group exhibited a smaller increase in anxiety intensity and struggle with anxiety in response to rising within-person levels of stress compared to those in the control group. In addition, we assessed whether they demonstrated a smaller decline in valued action under the same conditions. In this context, resilience is conceptualized as a dynamic, situation-specific ability to manage stress, with the intervention’s effectiveness being reflected in participants’ ability to adapt to context-specific stressors.

#### Statistical Analysis

##### Overview

The experiment design, exclusion criteria, hypotheses, and data analysis plan were preregistered [37]. The complete repository, including the preregistration, data analysis scripts, and data, is also available on the web [38]. Hypothesis testing and multiple comparison corrections were conducted in accordance with the preregistration. A power analysis was conducted for the longitudinal analysis to ensure an appropriate sample size for detecting the expected effects. However, the analysis of posttest and follow-up measures was not fully powered to detect small effects, which limits the conclusions that can be drawn from our analyses of those measures. Further details on the power analysis are provided in Multimedia Appendix 6.

We conducted a per-protocol analysis, including only participants who adhered to the compliance criteria specified in the inclusion and exclusion criteria. Participants who dropped out, experienced technical problems, did not meet participation criteria, or failed attention checks were excluded from the analysis as per protocol. This approach was chosen to ensure that the analysis reflected the effect of the intervention among those who followed the protocol as intended.

##### Pre- and Posttest and Follow-Up Analyses

We used analysis of covariance to compare the improvements in participants’ scores on 2 sets of questionnaires (well-being and mental health) between the groups. We assessed the effect of the experimental condition on each set of outcome variables using the following linear models:

Postintervention score ~ preintervention score + condition × scale

Follow-up score ~ preintervention score + condition × scale

These analyses control for individual differences before the test and integrate data from multiple measures while also acknowledging that the effect might differ between them. For post hoc comparisons, we applied the Bonferroni correction to control for multiple comparisons [39]. The power analysis for these analyses indicated that our sample size of 228 participants was underpowered to detect small-sized effects, with an achieved power of 0.32 (Cohen *f*=0.10, α=.05). As a result, caution should be taken when interpreting the findings, as small effects may not have been detected.

##### Longitudinal Data Analysis

To ensure sufficient statistical power for detecting the expected effects, a power analysis was conducted using Monte Carlo simulations in Mplus [40]. The analysis indicated that the sample size of 228 participants was adequately powered (power=0.9) for the longitudinal analyses, with a simulated dropout rate of 40%.

The data analysis followed the procedures outlined in the book by Bolger and Laurenceau [21]. We used an intensive longitudinal research design to model the within-participant causal process through which the intervention affects daily reactivity to stress in terms of affect and behavior. Our data analysis used a multilevel model. We predicted lower within-participant reactivity to stress in the treatment group compared to the control group.

In line with the recommendations, we opted for SAS PROC MIXED (SAS Institute) as our statistical analysis tool due to its ability to handle repeated measurements with unequal time intervals and effectively manage missing data. In addition, adhering to best practices, we distinguished between-participant and within-participant variations to accurately assess the impact of within-participant variation in stress. By focusing on within-participant variation, we can confidently eliminate any confounding effects of between-participant factors. Furthermore, we adopted a conservative approach in determining the df, using the number of participants (N=197) rather than the total number of observations. Finally, the variable time was rescaled such that 0 corresponds to the middle of the intervention period. A 1-unit difference in the variable time represents the passage of 1 day.

According to the preregistration, the hypotheses concerning the daily within-participant reactivity to stress, given the condition (within-participant stress *×* condition), are directional hypotheses. In contrast, the hypotheses regarding the general effect of condition on the outcome measures (condition) fall under the category of exploratory hypotheses, making them 2-tailed. To control the false discovery rate at a 5% level, we applied the Benjamini-Hochberg procedure [41] to all exploratory hypotheses.

## Results

### Overview

The Results section is structured into two main parts: (1) the intervention’s effect on mental health and well-being immediately after the intervention phase and 7 days later and (2) the longitudinal data on anxiety, emotional struggle, and valued action. We verified that there were no significant differences (all *P*>.30) between the control and treatment groups in terms of baseline demographic or psychological characteristics. Detailed baseline comparisons between groups across both categories are provided in Multimedia Appendix 7.

We provide additional details on the study’s dropout, compliance, engagement, and missing data and sparseness in Multimedia Appendix 8. This appendix outlines participant retention, compliance with study protocols, and an analysis of data sparsity, providing insights into participant engagement and potential data-related concerns.

**Figure 4:**
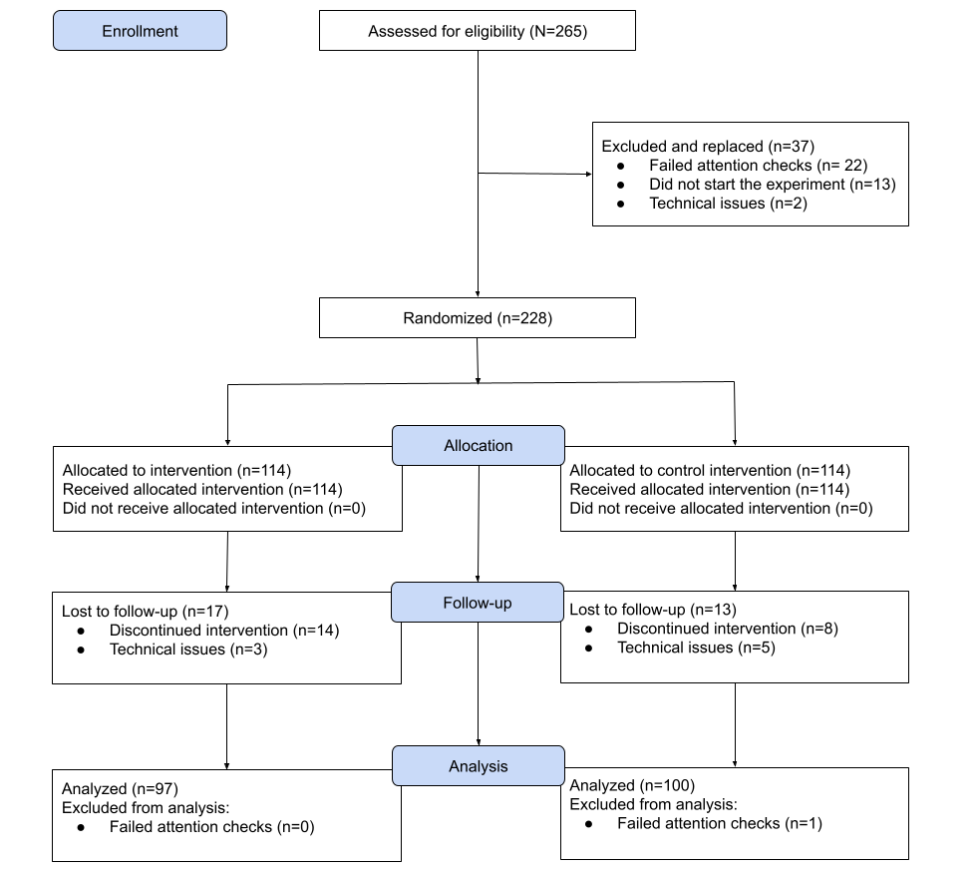
CONSORT (Consolidated Standards of Reporting Trials) flow diagram of the randomized controlled trial.

The CONSORT diagram illustrates the participant flow throughout the study, including enrollment, allocation, follow-up, and analysis phases (Figure 4). The diagram shows the enrollment of 228 participants, with 114 (50%) randomly allocated to the treatment group and 114 (50%) to a placebo control group. Attrition included 17 participants lost to follow-up in the treatment group and 13 in the control group. The final analysis comprised 97 participants in the treatment group and 100 in the control group.

### Mental Health and Well-Being After the Intervention

In this section, we present the results of the comparison between the treatment group and the control group at posttest and follow-up stages on participants’ scores related to mental health and well-being. This analysis examines the intervention’s effects on mental health and well-being while controlling for participants’ baseline scores before the intervention. Further details on the baseline anxiety sample characteristics, as well as the pre- and posttest means, SDs, medians, IQRs, please refer to Multimedia Appendix 9. This appendix provides a comprehensive overview of these metrics for each condition, time point, and anxiety-related variable.

The 2 groups’ posttest scores on measures of mental health neither significantly differed directly after the intervention (*F*_1,799_=0.173, *P*=.68, partial η^2^=0.0002) nor during the follow-up period (*F*_1,799_=0.235, *P*=.63, partial η^2^=0.0003). The posttest measures of mental health also did not significantly differ, neither directly after the intervention (*F*_1,799_=1.515, *P*=.22, partial η^2^=0.002) nor during the follow-up period (*F*_1,799_=0.719, *P*=.40, partial η^2^=0.0009).

### Longitudinal Data on Anxiety, Emotional Struggle, and Valued Action

#### Overview

**Figure 5:**
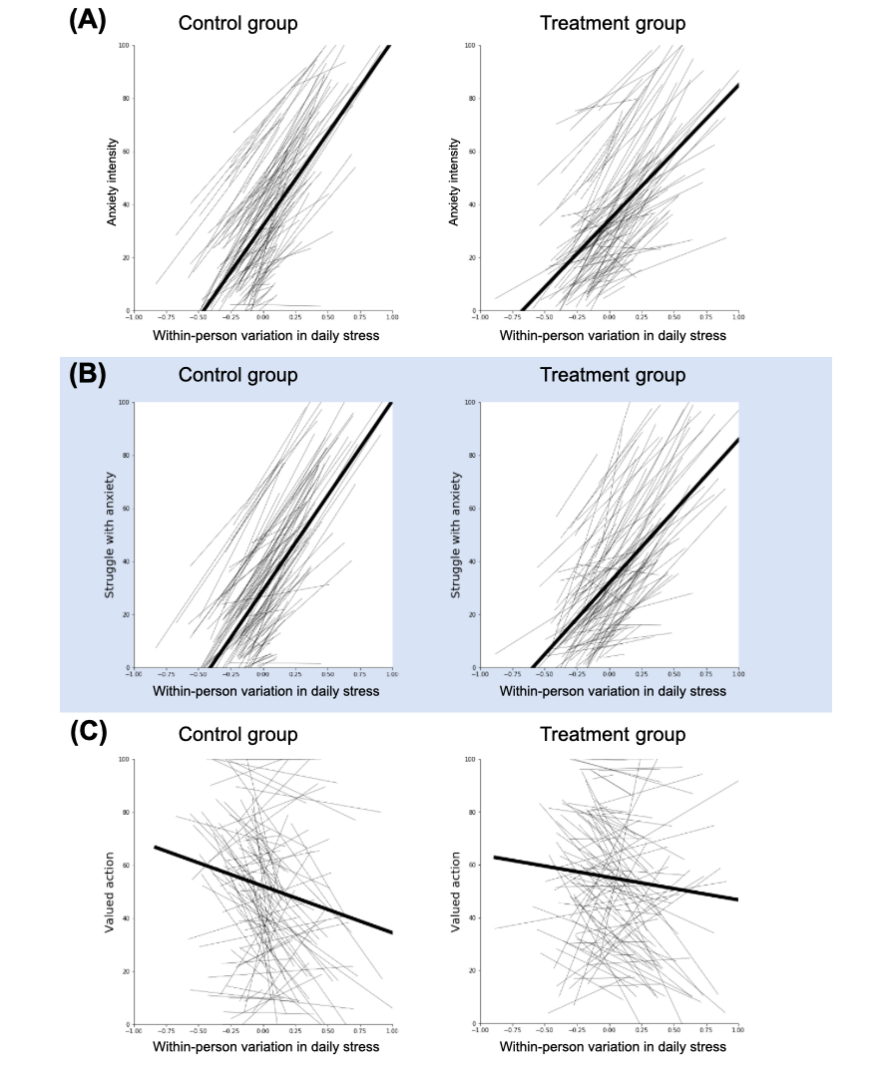
Spaghetti plots illustrating regression lines for individual participants (thin lines) and the average effect (thick lines). The panels present daily outcome measures in response to within-person variations in daily stress during the intervention period for the control and the treatment groups. Panel A depicts the intensity of anxiety, panel B shows the struggle with anxiety, and panel C displays intention enactment.

We used mixed-effects linear regressions to examine the effect of stress on participants’ daily scores of anxiety intensity, struggle with anxiety, and intention enactment during the 7-day intervention period. The spaghetti plots in Figure 5 illustrate the qualitative relationships between within-person variation in stress and daily scores for each outcome measure. Visually, the regression lines for participants’ anxiety intensity (Figure 5A) and struggle with anxiety (Figure 5B) appear to be less steep for the treatment group than the control group. This suggests that individuals in the treatment group exhibited lower reactivity to stress on both measures of affect. However, this does not appear to be the case for intention enactment (Figure 5C). It is also worth noting that intention enactment appears to be generally higher in the treatment group than in the control group.

In the subsequent sections, we present the results for each outcome measure separately. First, we report the main effect of group. Subsequently, to understand participants’ reactivity to stress during the intervention period, we analyze how the outcome variable varies because of a unit of increase in within-participant stress for each condition. Finally, we report the relative difference in reactivity to stress between groups.

#### Intensity of Anxiety

The regression analyses summarized in Table S1 in Multimedia Appendix 10 indicate that there was no significant main effect of condition on the average intensity of participants’ anxiety (2-tailed *t* test, *t*_197_=–1.62; *P*=.11; 95% CI –4.54 to 0.44) during the intervention period. In the control group, we found that the intensity of participants’ anxiety increased by 9 units (on a scale from 0 to 100) per unit of within-person variation in stress (*t*_197_=22.53; *P*<.001; 95% CI 8.21-9.78). In the treatment group, the intensity of participants’ anxiety increased by 7.64 units per unit of within-person variation in stress (*t*_197_=18.55; *P*<.001; 95% CI 6.83-8.46). This indicates that using InsightApp decreased reactivity to stress by 1.35 units of anxiety per unit of stress (1-tailed *t* test, *t*_197_=–2.38; *P*=.009; 95% CI –2.48 to 0.23). In relative terms, the treatment group’s level of anxiety was 15.05% less reactive to daily stressors than the control group’s level of anxiety.

#### Struggle With Anxiety

The regression analyses summarized in Table S2 in Multimedia Appendix 10 indicate that there was no significant main effect of condition on the degree to which participants struggled with anxiety (2-tailed *t* test, *t*_197_=–0.9; *P*=.37; 95% CI –3.76 to 1.4) during the intervention period. In the control group, we found that the strength of participants’ struggles with anxiety increased by 8.86 units of struggle per unit of within-person variation in stress (*t*_197_=22.1; *P*<.001; 95% CI 8.07-9.65). In the treatment group, the strength of participants’ struggles with anxiety increased by 7.79 units of struggle per unit of within-person variation in stress (*t*_197_=18.73; *P*<.001; 95% CI 6.97-8.61). This indicates that using InsightApp decreased the amount of emotional struggle the treatment group experienced in response to 1 unit of stress by 1.07 units per unit of stress (1-tailed *t* test, *t*_197_=–1.87; *P*=.031; 95% CI –2.2 to 0.06). In relative terms, for each unit of stress, the treatment group’s struggles with anxiety increased 12.09% less in response to stress than the control group’s struggles.

#### Valued Intention Enactment

The regression analyses summarized in Table S3 in Multimedia Appendix 10 show a significant general difference between conditions in the degree to which people enacted their valued intentions. On average, participants in the treatment condition enacted their valued intention 7.07 (0-100) points more than participants in the control condition (2-tailed *t* test, *t*_197_=3.23; *P*=.002; 95% CI 2.75-11.4). In the control group, the degree to which participants enacted the valued intentions decreased by 2.8 units per unit of within-person variation in stress (*t*_197_=–3.77; *P*<.001; 95% CI –4.26 to 1.34). In the treatment group, the degree to which participants enacted the valued intention in the treatment condition decreased by 1.75 units (*t*_197_=–2.3; *P*=.02; 95% CI –3.25 to –0.25) per unit of within-person variation in stress. The interaction between stress and condition had no significant effect on intention enactment (1-tailed *t* test, *t*_197_=1.0; *P*=.16; 95% CI –1.03 to 3.13).

The autocorrelation (Tables S1-S3 in Multimedia Appendix 10) of the data was statistically significant for the intensity of participants’ anxiety (SP POW=0.102; *z* score=2.31; *P*=.04; 95% CI 0.016-0.189) and how much they struggled with anxiety (SP POW=0.13; *z* score=0.93; *P*=.002; 95% CI 0.052-0.22). However, the magnitude of these coefficients indicates only a weak positive autocorrelation in the level 1 (ie, within-participant) residuals. As shown by the magnitude (0.039) and *P* value of the coefficient (*P*=.45), there is no evidence of autocorrelation for the case of valued action.

We ran the same analysis for the 7-day postintervention period. The results indicated that the degree to which the treatment group struggled with anxiety in response to 1 unit of stress was still 1.43 units lower than that of the control group (*t*_197_=–2.84; *P*=.002; 95% CI –2.42 to 0.44). In other words, even after the support of InsightApp was removed, each unit of stress increased the treatment group’s struggle with anxiety <18% of the control group’s struggle with anxiety. We found no other significant differences between the 2 conditions during the postintervention period (Multimedia Appendix 11).

Finally, to determine whether baseline anxiety levels influenced the effectiveness of the intervention, we conducted exploratory analyses. After incorporating relevant interaction terms into our models, we found that baseline anxiety traits were not statistically significant (all *P*>.05), either during the intervention or in the postintervention phase. Consequently, we cannot conclude that baseline anxiety traits moderated the intervention’s effectiveness. Details of the exploratory analysis have been provided in Multimedia Appendix 10.

## Discussion

### Principal Findings

In this randomized controlled trial, we examined the effectiveness of InsightApp in helping individuals cope with stress and anxiety in real-life settings. During the intervention period, the treatment group was significantly more resilient to stress in terms of the intensity of their anxiety and the degree to which they struggled with it. Moreover, the treatment group demonstrated a higher enactment of their valued intentions compared to the control condition. These effects were sustained in the postintervention period regarding the struggle with anxiety. However, the postintervention scores and follow-up scores on measures of well-being and mental health did not significantly differ between conditions (all *P*>.22).

### Limitations

The experiment had the following limitations. First, the sample was relatively homogeneous, lacking diversity in ethnicity, education, and location. This limits the generalizability of findings across different cultural backgrounds. In addition, as the sample was drawn from the general population, the intervention’s effectiveness may vary for individuals with higher anxiety levels. Participants were also compensated, potentially affecting their engagement and motivation, which could differ from those in the general population without such incentives.

Second, the relatively brief training and postintervention period (ie, 7 days) limit our understanding of the intervention’s long-term effectiveness. Extended use may yield more pronounced effects. The duration of sustained improvements beyond the 7-day postintervention phase is also uncertain. Furthermore, the results of our longitudinal data analysis should be taken with a grain of salt because some time series were weakly autocorrelated. Third, the experiment design has room for improvement. Presurvey measures may have unintentionally provided psychoeducation to the control group. Moreover, as the control condition included elements of the reflection modules, such as the reactivity and values modules, our results may underestimate the benefits of the complete InsightApp as a package intervention.

Two significant limitations impacted the pre-post measurement of anxiety in this study. First, the study was underpowered (power of 32.4%, *f*=0.10, α=.05) to detect small effects on posttest and follow-up measures of psychological traits, and because our intervention was short, small effects were to be expected. Therefore, the nonsignificant findings do not imply that the corresponding effects do not exist. Rather, our study was underpowered to conclusively determine the significance of long-term effects on psychological traits**.**

Furthermore, the administration of the HAM-A posed challenges. This scale was administered via an online self-report format rather than the traditional clinician-administered interview. The HAM-A is typically conducted by a trained clinician to ensure an accurate interpretation of the items, which may not be as intuitive for participants to self-assess. Although we provided clear instructions, the absence of direct clinician oversight may have affected the reliability and validity of the results. This deviation from the standard administration method should be considered when interpreting the findings, as it may introduce variability in how participants understood and responded to the scale.

Another limitation of this study is the brief duration of the intervention, which may have restricted the ability to detect changes in trait-level measures of anxiety that typically require longer periods to show. Extending the intervention beyond 7 days may enhance its impact, particularly on more stable, trait-like outcomes, and should be explored in future research.

Another potential concern that could affect the interpretability of the results is the evaluation of the app in a population that may not have fully endorsed anxiety symptoms. To address this concern, we conducted an analysis of baseline anxiety levels, as detailed in Multimedia Appendix 9, specifically in the Anxiety Sample Characteristics section. This analysis shows that even though we recruited our sample from the general population, it included a broad spectrum of anxiety levels, with >70% of participants reporting moderate to high anxiety traits. There was no evidence that the effectiveness of the intervention varied for participants with differing baseline levels of anxiety (*P*>.05; refer to Multimedia Appendix 9). However, because the app was not tested in a clinical sample, we cannot conclude that the intervention would be effective or have a similar size effect in a clinical population. This makes testing the intervention in a clinical sample an important direction for future research.

Finally, while we observed significant effects in the longitudinal data, we did not find such significant results in the comparison of pre- versus posttest measures. The lack of pre-post significant results could raise questions regarding the overall impact of the intervention on participants’ psychological well-being. In the subsequent section, we explore potential factors that could explain the lack of statistically significant pre-post effects.

### Interpretations

The intervention effectively reduced daily anxiety levels and struggle with anxiety compared to the control group. However, no statistically significant differences were observed in postintervention self-report questionnaires. When interpreting these results, it is important to consider that this discrepancy may stem from the study’s short duration and its lack of power to detect small effects in pre-post measures. In addition, differences in outcome variables could also contribute to these findings. The experience sampling data showed anxiety reduction in specific stress situations, while postintervention questionnaires assessed general mental health and well-being regardless of stress. In addition, although the wording of the instructions for the self-report measures—originally designed to assess traits—was adjusted to reflect the past two weeks, it may still have lacked sensitivity to detect short-term intervention effects. Longer interventions may have a more substantial impact on traits, whereas shorter-term measures, such as ecological momentary assessment or daily diaries, are more sensitive to short-term fluctuations in psychological states [20,42]. Further investigation and extended interventions are needed for a comprehensive understanding of the intervention’s effectiveness in promoting psychological well-being.

Notably, participants’ improvement in anxiety struggle levels during the intervention was sustained and even reduced after the intervention. However, the effect on anxiety intensity did not persist. This suggests that the app’s focus on metacognitive skills may help users acknowledge and accept intense emotions, reducing their struggle with anxiety even without app support. However, participants might have relied on the app for anxiety intensity reduction rather than the app’s general strategies. Further investigation is required to understand how the app impacted anxiety intensity and develop strategies for users to experience these benefits independently of the app. Differential retention of effects between anxiety intensity and struggle underscores their distinct dimensions in an individual’s anxiety experience. Measuring and addressing both aspects is crucial for a comprehensive understanding of anxiety’s impact on well-being.

Another point worth noting when interpreting the results is that baseline anxiety traits did not significantly moderate the intervention’s outcomes. This indicates that there is no evidence to suggest that the intervention’s effectiveness varies across different levels of baseline anxiety. Although the app was tested on the general population, the sample exhibited a broad range of anxiety levels, with a substantial proportion of participants experiencing moderate to high anxiety traits, underscoring broad applicability.

### Comparison With Prior Work

In this section, we compare the results of our experiment with previous research in the field. InsightApp collects data using experience sampling and pre-post follow-up assessments of mental health and well-being. Most studies on EMIs for anxiety only rely on pre-post comparisons [9,43,44]. By contrast, our experiment additionally used experience sampling to track participants’ reactivity to stress on a daily basis during and after the intervention. To the best of our knowledge, there is currently no other experiment that examined how EMIs moderate the effects of stress on daily reports of anxiety, struggle with anxiety, and intention enactment. Furthermore, InsightApp incorporates multiple psychotherapeutic strategies from ACT, CBT, and mindfulness-based cognitive therapy into an integrated gamified approach. To the best of our knowledge, previous mental health apps for stress and anxiety have not integrated all of these components into a game for people to practice metacognitive skills with their real-life challenges. Therefore, we compare our findings with previous studies on digital interventions that include ≥1 component similar to those of InsightApp.

Mindful awareness and acceptance of emotions are key components actively trained by InsightApp. Previous studies in the literature on stress coping have provided evidence supporting the idea that being aware of the present moment can reduce emotional reactivity to stressors [6,7,45-47]. Our results indicate that when participants engage in a daily practice of noticing, accepting, and embracing their emotions, they experience significantly less anxiety when facing stressors and report fewer struggles with their feelings of anxiety compared to the control group. Our findings highlight the potential benefits of incorporating these practices into daily life beyond the controlled settings of a laboratory environment. In addition, our experiment supports the notion that training metacognitive skills with daily challenges leads to sustained improvements. The findings indicate that metacognitive skills for coping with stress and anxiety are trainable and can be effectively improved with a relatively small amount of practice. Importantly, the positive effects of this training on the degree to which participants struggle with anxiety were sustained after the training period ended. This suggests that metacognitive skills can be cultivated and applied in everyday life. This makes them valuable tools for managing stress and anxiety.

Some studies have also examined the interaction between mindfulness, coping with stress, and value-congruent action. These studies have found evidence supporting the hypothesis that individuals who possess higher levels of present-moment awareness are less emotionally reactive to stressors and are more likely to engage in value-congruent actions [17,46-48]. The results of our experiment consistently align with previous findings. Participants who practiced awareness and acceptance of their emotions using InsightApp demonstrated significantly lower emotional reactivity to stressors, experienced less anxiety, and exhibited a higher frequency of enacting their valued intentions compared to the control group.

As reported in the Results section, participants’ scores on postintervention measures of mental health and well-being did not show significant improvement compared to the control group, neither directly after the 7-day intervention phase nor in the follow-up assessments. The meta-analysis conducted by Versluis et al [43] covered 15 interventions targeting anxiety, indicating a small to medium effect size of Hedges *g*=0.47 on pre- and posttest anxiety measures.

When contrasting the effectiveness of InsightApp with these interventions, it is important to take into account factors such as the intervention duration, experimental design, the inclusion of control conditions, and the strength of those control conditions. As discussed in the Methods section, previous work has emphasized the significance and complexities involved in crafting robust control conditions that can effectively address the digital placebo effect [22,23]. This phenomenon involves improvements in outcome variables that are driven by their belief in the effectiveness of the digital intervention. To effectively address the digital placebo effect, control conditions should closely resemble the treatment condition. Hence, it is crucial for them to exhibit structural equivalence and similar aesthetics, quality, and usability as the experimental condition [23]. In addition, the control condition should feel credible to participants, and the description of the benefits that participants can expect from participating in the experiment should be identical. Waitlist controls fail to address the placebo effect, as participants do not anticipate receiving benefits between assessments. Consequently, studies with control conditions dissimilar to the treatment intervention or waitlist controls might overestimate the intervention’s impact on participants. Experiments with strong control conditions are still rare in research on EMIs for anxiety. For example, from the 15 interventions targeting anxiety reviewed by Versluis et al [43], only 7 included a control group. Of those interventions, only 4 incorporated a placebo control groupor an active control group. Therefore, the effect size of *g*=0.47 might overestimate the real effect of the digital interventions on participants’ pre-post measures of anxiety. Our experiment addressed this problem by using an active placebo control group that is highly isomorphic to the treatment group. Our experiment thereby isolated the active component under examination. Moreover, our experimental design was attuned to participants’ improvements in daily life. This strategy helped to prevent the potential overestimation of the intervention effects in pre-post outcome measures while also allowing for the capture of more nuanced and gradual improvements in participants’ mental health and well-being.

Finally, the intervention presented in this study was tested as an intensive, short-term treatment, with 1 session per day over a week. The complete study lasted 3 weeks, including a pre- and postintervention period. This contrasts with traditional psychotherapy, which typically spans several weeks or months but usually involves only 1 session per week. Unlike in therapy, short, intensive interventions are not uncommon in research on EMIs. For example, a systematic review of 19 papers on EMIs reported that study durations ranged from 2 to 15 weeks, with an average of 4 weeks [49]. Another study evaluating 26 EMI studies based on CBT principles reported that study durations ranged from 2 weeks to 6 months, with a median duration of 30 days [50].

### Conclusions

In this experiment, we conducted a randomized controlled trial combining 2 distinct experimental designs: an experience sampling design and a pre-post follow-up design, each applied to different outcome measures. The experiment’s results showed significant improvements in participants’ anxiety, struggle with anxiety, and intention enactment as reactivity to stress on momentary daily measures. Moreover, the improvement in participants’ struggle with anxiety persisted after the end of the intervention, indicating that participants internalized at least some of the emotion regulation strategies InsightApp had directed them to use during the intervention. However, these positive changes did not lead to significant improvements in self-report measures of overall well-being and mental health. Further research is needed to resolve the apparent contradictions between the observed improvements during the training and the lack of improvements in pre-post measures of general mental health. Future studies should explore intervention durations longer than those used in this study and be adequately powered to detect small improvements, ensuring that subtle effects are not missed due to insufficient sample sizes.

## Appendix

Multimedia Appendix 1 The InsightApp.

Multimedia Appendix 2 Inclusion and exclusion criteria and recruitment timeline.

Multimedia Appendix 3 Demographic baseline characteristics by group.

Multimedia Appendix 4 CONSORT (Consolidated Standards of Reporting Trials) checklist.

Multimedia Appendix 5 Placebo control.

Multimedia Appendix 6 Randomization, blinding process, and power analysis.

Multimedia Appendix 7 Baseline comparison between groups.

Multimedia Appendix 8 Dropout, compliance, engagement, and missing data and sparseness.

Multimedia Appendix 9 Baseline characteristics and pre-post and follow-up assessments of psychological measures.

Multimedia Appendix 10 Results tables for intervention period and exploratory analysis.

Multimedia Appendix 11 Results for the postintervention period.

## Abbreviations

ABCDE: activating event, belief system, consequences, disputation of beliefs, and effective new beliefs
ACT: acceptance and commitment therapy
CBT: cognitive behavioral therapy
CONSORT: Consolidated Standards of Reporting Trials
EMI: ecological momentary intervention
HAM-A: Hamilton Anxiety Rating Scale

## Data Availability

The datasets generated and analyzed during this experiment are available in Open Science Framework repository [37].
