# Supplementary material for: Evaluating the Effectiveness of InsightApp for Anxiety, Valued Action, and Psychological Resilience: Longitudinal Randomized Controlled Trial
Source: JMIR Ment Health. 2025 Feb 4;12:e57201. doi: 10.2196/57201 (PMC11836588; doi:10.2196/57201)
Supplement: Multimedia Appendix 1 [file mental_v12i1e57201_app1.docx]

Multimedia Appendix 1 - The InsightApp

Table S1: Summary of questions included in the Reactivity Module

| Strategy | Question | Answer type |
| --- | --- | --- |
| CBT - Step A (Activating event) | Which recurring situation in your daily life makes you feel the most anxious? | Open text entry |
| CBT - Step A (Activating event) | How difficult or problematic is the situation in the context of your life? | 0-10 |
| CBT - Step A (Activating event) | Please give the situation a descriptive title | Open text entry |
| CBT - Step A (Activating event) | To which category does the situation belong? | Multiple choice |
| CBT - Step A (Activating event) | How often do you meet this situation | Multiple choice |
| CBT - Step A (Activating event) | When did this particular situation start? | Multiple choice |
| CBT - Step A (Activating event) | In total, how long may the situation last? | Multiple choice |
| CBT - Step C (consequences) | How often do you feel anxious? | Multiple choice |
| CBT - Step C (consequences) | How intense does anxiety feel right now? | Slider (0-100) |
| CBT - Step C (consequences) | With *struggle*, we mean the degree to which you suffer because of the emotion. How strong is the struggle with anxiety right now? | Slider (0-100) |
| CBT - Step C (consequences) | Which unwanted behavior or action arises when you feel anxious? When I feel anxious, then you tend to… | Multiple choice with example answers or open text entry |
| CBT - Step C (consequences) | To which extent did you [unwanted action] last week? | Multiple choice with example answers or open text entry |
| CBT - Step C (consequences) | How likely are you to [unwanted action] in your current emotional state? | Slider (0-100) |

Table S2. Summary of questions and information included in the Valued Action Module

| ACT - values | From the list, which values and virtues would you like to express in your current situation?  <List of Values and Virtues> multimedia appendix x | Multiple picks from a list of 30 values and virtues. |
| --- | --- | --- |
| ACT - Committed action | List of selected values is displayed on the top  In your current situation, which way of acting aligns best with your values? | Multiple choice with example answers or open text entry (char x-y) |
| ACT - Committed action | To which extent did you [valued action] last week? | Slider (0-100) |
| ACT - Committed action | How likely are you to [valued action] in your current emotional state? | Slider (0-100) |

Table S3. Summary of questions and information included in the Choice Point Module

| Components | Text |
| --- | --- |
| Main screen | What are you doing next?  Away move: Strengthen the old and reactive habit.  Towards move: Strengthen the new and valued habit. |
| Info button | Title: Choice point!  Text: A choice point is a moment of awareness, in which you notice that an emotion and a tendency to react to that emotion is active. By accepting and witnessing the emotion and the impulse to act without being taken over by them , you have the choice of breaking that old habit and building a new one. |
| Towards move | Title: Towards move!  Message: You have chosen to give your best at enacting your valued action instead of the old unwanted action.  Button: I can do it! |
| Away move | Title: Away move  Message: Observe the process of giving into the old unwanted action with self-compassion and without indulging. Remember that every moment gives you a new chance to choose.  Button: I’ll do that |

Table S4. Summary of Questions and Information in the Metareasoning Coach

| Strategy | Question | Answer type |
| --- | --- | --- |
| CBT - Step B (Beliefs) | In your current situation, when you feel [emotion], which thought goes through your mind? | Example answer multiple choice or open text entry |
| CBT - Step B (Beliefs) | Which belief might be underlying those thoughts? | Multiple choice with example answers or open text entry |
| CBT - Step B (Beliefs) | How much do you believe in it? | 0-100 |
| CBT - Step C  (Consequences) | Believing [unhelpful belief] makes you feel anxious and tend to [unwanted action] | Summary text of previous steps. |
| Disidentification aid | If anxiety would have a color, which color would it be? | Multiple choice |
| Disidentification aid | And if anxiety would have a form? | Body and hand position choice |
| Disidentification aid | Give it a funny name! | Multiple choice with example answers or open text entry |
| Disidentification aid | Please play with the slider to get to know [avatar name] | Animated change in facial expression of avatar controller by slider |
| Gamification | You caught [avatarName]!  Every time you notice and embrace little monsters, you grow the light of your awareness and collect *Insight Points.* | Introduction to *Insight Point* rewards. |
| CBT - Step D  (Dispute) | The unhelpful belief is displayed on the top.  Please add examples from your experience that contradict this belief. | Open text entry |
| CBT - Step D  (Dispute) | What would you say to a loved one, if they feel this way? | Open text entry |
| CBT - Effective approach | Which belief encourages you to [valued action]? | Multiple choice with example answers or open text entry |
| CBT - Step C  (Consequences) | How does [helpful belief] make you feel? | Multiple choice |
| CBT - Step C  (Consequences) | Believing [unhelpful belief] makes you feel [positive emotion] and tend to [valued action] | Summary text of previous steps. |
| CBT - Step C  (Consequences) | Chose the belief that brings you closer to your goal | Switch choice |

Table S5. Characteristics of the Ecological Momentary Intervention delivered by the InsightApp.

| Category | Specification |
| --- | --- |
| Platform type | The InsightApp is an iOS app designed for smartphones. |
| EMI type | The InsightApp is an Interactive Ecological Momentary Intervention (EMI). It stores participants' inputs and customizes the app's content, messages, and presentations according to individual user data. |
| EMA surveys | Morning practice  Cognitive defusion practice  Evening report |
| EMA response scales | The app utilizes single-item sliding scales ranging from 0 to 100 and multiple-choice questions for EMA surveys. |
| EMA duration (days of data collection) | Morning practice: 7 days  Cognitive defusion practice: 7 days  Evening report: 18 days |
| Temporal contextualizations | Morning practice: Momentary contextualization (at the moment)  Cognitive Defusion Practice: Momentary contextualization (at the moment)  Evening Report: Daily contextualization (for the entire day) |
| Pings per day | Morning practice: One daily reminder.  Cognitive defusion practice: Two random reminders.  Evening report: One daily reminder. |
| Reliability and validity | We used the measure of single-item sliding scales from Amo., et al, 2023, for momentary and daily levels of anxiety, struggle with anxiety, unwanted action, valued action, and the endorsement of the unhelpful and helpful beliefs [1]. |

## References

[1] Amo V, Prentice M, Lieder F. A gamified mobile app that helps people develop the metacognitive skills to cope with stressful situations and difficult emotions: formative assessment of the InsightApp. JMIR Form Res 2023 Jun 16; 7:e44429
