# Supplementary material for: Evaluating the Effectiveness of InsightApp for Anxiety, Valued Action, and Psychological Resilience: Longitudinal Randomized Controlled Trial
Source: JMIR Ment Health. 2025 Feb 4;12:e57201. doi: 10.2196/57201 (PMC11836588; doi:10.2196/57201)
Supplement: Multimedia Appendix 2 [file mental_v12i1e57201_app2.docx]

Multimedia Appendix 2 - Inclusion and Exclusion Criteria, and Recruitment Timeline

Inclusion and Exclusion Criteria

**Inclusion Criteria:** We recruited a balanced sample of English-speaking (from the US and UK), and iPhone-owning adults on Prolific. To be allowed in the study, participants had to give consent and must not have participated in previous studies delivered by the InsightAppExperiment.

**Exclusion Criteria:** Participants who failed the attention checks or failed to meet the participation criteria were excluded from the experiment.

**Attentional Checks:** We measured if participants were answering questions at random by defining a minimum amount of time needed to read and answer a question. Participants who answered more than 10 to 30 questions (varies depending on the set of questionnaires) in less than 1.6 seconds failed the attention check.

- **Onboarding surveys:** Participants were directly excluded after failing the attentional check.
- **Middle surveys:** Participants had three opportunities to pass the attention checks.
- **Offboarding surveys:** Participants had three opportunities to pass the attention checks.

**Participation Criteria:** Participants were asked to participate in the study daily. We excluded participants who did not interact with the app for a minimum number of days as follows:

- **Stage 0** (4 days long): Participants had to complete at least two evening reports.
- **Stage 1** (7 days long): On at least three days, participants had to complete both the evening reports and the morning practice.
- **Stage 2** (7 days long): Participants had to complete at least three evening reports.

Participants that were excluded for taking too long to complete a transition step between stages included:

- **Onboarding:** Participants had a maximum of one day to finish the complete onboarding (the same day they joined the study).
- **Introduction to Morning Practice:** Participants had a maximum of two days to finish the introduction.
- **Mid-Surveys:** Participants had a maximum of three days to finish the mid-surveys.
- **Offboarding:** Participants had a maximum of three days to finish the offboarding.

## Recruitment and Data Collection Timeline

Enrollment for our study was conducted at two distinct time points to ensure a full participant cohort after initial exclusions and dropouts that occurred before the intervention in the control and experimental groups began to differ on day 5. The first round of enrollment occurred on 10 October 2022 and concluded the same day. After assessing initial exclusions and dropouts, a second enrollment phase took place on 19 October 2022 to replace participants, also concluding that day. Overall, data collection spanned from 10 October 2022, when the first group of participants began onboarding, to 6 November 2022, when the second group completed offboarding. Table S1 presents a detailed breakdown of the main tasks and their timing for each round of participants.

Table S1. Participants Recruitment and Data Collection Timeline

| **Day** | **Task** | **Stage** | **Recruitment round 1** | **Recruitment round 2** |
| --- | --- | --- | --- | --- |
| 1 | Onboarding & payment code 1  Evening report | 0 | 10 October 2022  10 October 2022 | 19 October 2022 |
| 2 | Evening report | 0 | 11 October 2022 | 20 October 2022 |
| 3 | Evening report | 0 | 12 October 2022 | 21 October 2022 |
| 4 | Evening report | 0 | 13 October 2022 | 22 October 2022 |
| 5 | Introduction to morning practice + evening report | 1 | 14 October 2022 | 23 October 2022 |
| 6 | Morning practice + evening report | 1 | 15 October 2022 | 24 October 2022 |
| 7 | Morning practice + evening report | 1 | 16 October 2022 | 25 October 2022 |
| 8 | Morning practice + evening report | 1 | 17 October 2022 | 26 October 2022 |
| 9 | Morning practice + evening report | 1 | 18 October 2022 | 27 October 2022 |
| 10 | Morning practice + evening report | 1 | 19 October 2022 | 28 October 2022 |
| 11 | Morning practice + evening report | 1 | 20 October 2022 | 29 October 2022 |
| 12 | Mid-study surveys + evening report | 2 | 21 October 2022 | 30 October 2022 |
| 13 | Evening report | 2 | 22 October 2022 | 31 October 2022 |
| 14 | Evening report | 2 | 23 October 2022 | 1 November 2022 |
| 15 | Evening report | 2 | 24 October 2022 | 2 November 2022 |
| 16 | Evening report | 2 | 25 October 2022 | 3 November 2022 |
| 17 | Evening report | 2 | 26 October 2022 | 4 November 2022 |
| 18 | Evening report | 2 | 27 October 2022 | 5 November 2022 |
| 19 | Offboarding & payment code 2 |  | 28 October 2022 | 6 November 2022 |
