# Supplementary material for: Evaluating the Effectiveness of InsightApp for Anxiety, Valued Action, and Psychological Resilience: Longitudinal Randomized Controlled Trial
Source: JMIR Ment Health. 2025 Feb 4;12:e57201. doi: 10.2196/57201 (PMC11836588; doi:10.2196/57201)
Supplement: Multimedia Appendix 3 [file mental_v12i1e57201_app3.docx]

Multimedia Appendix 3 - Demographic Baseline Characteristics by Group

Table S1 presents the baseline demographic characteristics of participants in both the control and experimental groups. The age distribution was comparable between groups, with the control group having an average age of 39.14 years (Std = 12.96, Median = 36.5, IQR = 16.25), while the experimental group had an average age of 37.58 years (Std = 9.85, Median = 37, IQR = 12.25). The gender distribution showed approximately equal proportions of male and female participants across both groups. The majority of participants identified as White, with smaller proportions represented in other ethnic groups. Most participants resided in the United Kingdom, with a small number from the United States. Employment status was also similar across groups, with the majority employed, followed by students and unemployed participants.

Table S1. The variables include continuous measures such as age, as well as categorical variables like gender, ethnicity, country of residence, and employment status. For continuous variables such as age, we report the mean, standard deviation (Std), median, and interquartile range (IQR) to provide a comprehensive view of the data distribution. Categorical variables are presented with counts and percentages (Pct) to highlight the distribution of participants across different demographic categories.

| **Variable** | **Condition** | **Category** | **Count** | **Pct** | **Mean** | **Std** | **Median** | **IQR** |
| --- | --- | --- | --- | --- | --- | --- | --- | --- |
| Age | Control | - | - | - | 39.14 | 12.96 | 36.5 | 16.25 |
|  | Experimental | - | - | - | 37.58 | 9.85 | 37 | 12.25 |
| Sex | Control | Female | 50 | 52.10% | - | - | - | - |
|  |  | Male | 46 | 47.90% | - | - | - | - |
|  | Experimental | Female | 51 | 53.70% | - | - | - | - |
|  |  | Male | 45 | 46.30% | - | - | - | - |
| Ethnicity | Control | Asian | 2 | 2.08% | - | - | - | - |
|  |  | Black | 2 | 2.08% | - | - | - | - |
|  |  | Mixed | 2 | 2.08% | - | - | - | - |
|  |  | Other | 0 | 0.00% | - | - | - | - |
|  |  | White | 90 | 93.75% | - | - | - | - |
|  | Experimental | Asian | 5 | 5.21% | - | - | - | - |
|  |  | Black | 1 | 1.04% | - | - | - | - |
|  |  | Mixed | 3 | 3.13% | - | - | - | - |
|  |  | Other | 2 | 2.08% | - | - | - | - |
|  |  | White | 85 | 88.54% | - | - | - | - |
| Country of Residence | Control | United Kingdom | 115 | 98.31% | - | - | - | - |
|  |  | United States | 2 | 1.69% | - | - | - | - |
|  | Experimental | United Kingdom | 96 | 95.83% | - | - | - | - |
|  |  | United States | 5 | 4.17% | - | - | - | - |
| Employment | Control | Employed | 69 | 70.87% | - | - | - | - |
|  |  | Student | 13 | 12.62% | - | - | - | - |
|  |  | Unemployed | 17 | 16.50% | - | - | - | - |
|  | Experimental | Employed | 73 | 70.41% | - | - | - | - |
|  |  | Student | 17 | 17.35% | - | - | - | - |
|  |  | Unemployed | 12 | 12.24% | - | - | - | - |
