# Supplementary material for: Evaluating the Effectiveness of InsightApp for Anxiety, Valued Action, and Psychological Resilience: Longitudinal Randomized Controlled Trial
Source: JMIR Ment Health. 2025 Feb 4;12:e57201. doi: 10.2196/57201 (PMC11836588; doi:10.2196/57201)
Supplement: Multimedia Appendix 5 [file mental_v12i1e57201_app5.docx]

Multimedia Appendix 5 - Placebo control

In this section, we describe the design of the placebo control condition and how it addresses emotion goals, demand characteristics, and the digital placebo effect.

To control emotion goals and demand characteristics, both conditions shared the same emotional and behavioral goals and context. Participants in both groups reflected on challenging situations, rated anxiety intensity, identified typical reactions, considered values, and aimed to improve behavior under stress (refer to *Reactivity* and *Values Module*). The placebo intervention thereby controlled for the emotion goal effect. Furthermore, the shared context and goals shared across both groups create similar expectations regarding the researchers’ objectives, thereby controlling for demand characteristics. The placebo condition differed from the treatment only in the specific components under study. The treatment group worked with the meta-awareness and meta-reasoning coach, while the control group answered questions about psychological traits, preferences, and attentional skills (see Figure S1.A and S1.B). For a full list of control group questions, refer to Table S1. Additionally, the placebo control group replaced the meta-awareness coach with a Stroop task (Figure S1.C) and a spatial memory task (Figure S1.D) sourced from the ResearchKit (Apple Inc) library [1]. These tasks enhance cognitive control and attentional skills linked to improved well-being [2-4].

To control for the digital placebo effect, both groups were blinded to their treatment allocation and received identical information about the experiment's purpose and the expected benefits of the InsightApp. Both were informed that the InsightApp aimed to enhance metacognitive and cognitive skills for managing daily stress. The control group also received credible metacognitive and cognitive exercises commonly used in executive functions training programs and commercial brain training apps [5-7]. These tasks logically aligned with the experiment's framework, thereby controlling for the digital placebo effect. Furthermore, the placebo control intervention closely mirrored the structure of the treatment intervention (isomorphism). Both interventions used the same mobile platform and interface, ensuring identical quality, aesthetics, and usability. The experiment timeline, session durations, and the number of daily formal training sessions and assessments (morning practices and evening reports) were the same in both conditions. The only structural difference was that the treatment group used the InsightApp during the day to embrace their emotions, resulting in an average of 2 extra minutes of daily app interaction. This high structural equivalence enhances the placebo control's effectiveness in managing the digital placebo effect.


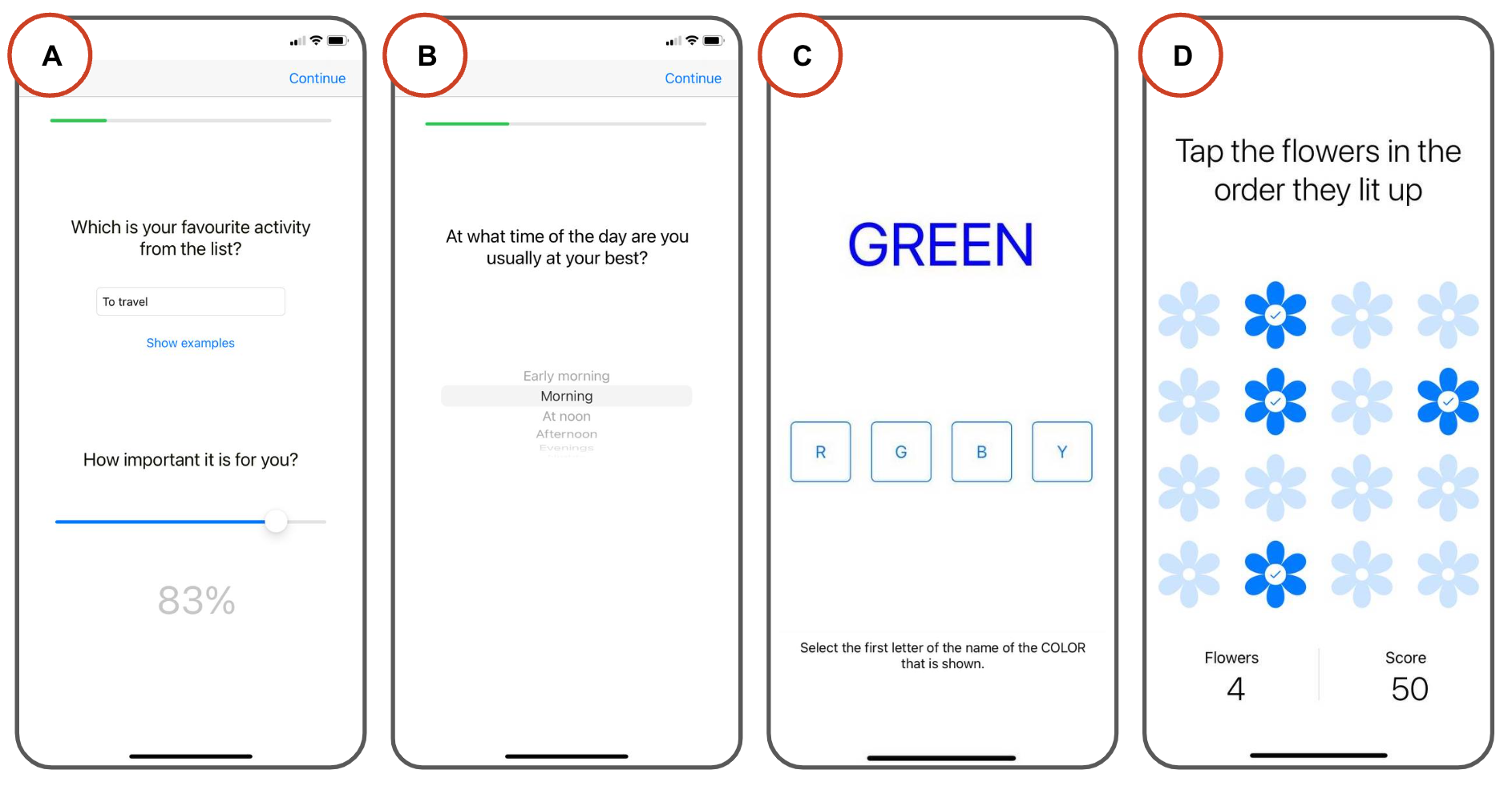


Figure S1. Screenshots from the control condition’s tasks. Panels A-B show example screens illustrating how the app guides users to answer unrelated questions regarding their preferences. Panel C shows the Stroop task, and panel D shows the spatial memory task.

Table S1: Summary of questions included in the placebo control reflection tasks

| Strategy | Question | Answer type |
| --- | --- | --- |
| Instructions | In this exercise, you will reflect on and answer a series of questions regarding yourself.  Please ensure you are in a quiet place and won’t be interrupted during the reflection. |  |
| Personality | How do you perceive your level of extraversion? | Multiple choice |
| Preferences | Which is your favorite activity from the list?  How important is it for you? | Slider 0-100 |
| Personality | At what time of the day are you usually at your best? | Multiple choice |
| Preferences | Which color do you prefer? | Multiple choice |
| Preferences | Who do you prefer to spend time with?  Please list reasons why you mostly prefer spending time with [selected choice] | Multiple choice  Open text entry |
| Planing task | Please plan a weekend trip using 3 to 5 bullet points. | Open text entry |
| Personality | In which area of your life do you currently experience the most uncertainty?  How comfortable do you feel about change in this area? | Slider 0-100 |
| Preferences | Which skills would you prefer to have? | Multiple choice |
| Attention task | What is the correct email address of Carl? | Multiple choice |
| Attention task | What is the correct email address of Angelina? | Multiple choice |
| Attention task | What is the correct telephone number of Jesse? | Multiple choice |
| Attention task | What is the correct telephone number of Bob? | Multiple choice |

## References

[1] Apple's ResearchKit frees medical research. Nat Biotechnol 2015 Apr 7; 33:322

[2] Kryla-Lighthall N, Mather M. The role of cognitive control in older adults' emotional well-being. In: Bengston VL, Gans D, Pulney NM, Silverstein M, editors. Handbook of Theories of Aging. Cham, Switzerland: Springer; 2009.

[3] Ochsner KN, Gross JJ. The cognitive control of emotion. Trends Cogn Sci 2005 May; 9(5):242-9

[4] Robinson MD, Eid M. The Happy Mind: Cognitive Contributions to Well-Being. Cham, Switzerland: Springer; 2017.

[5] Titz C, Karbach J. Working memory and executive functions: effects of training on academic achievement. Psychol Res 2014 Nov; 78(6):852-68

[6] Diamond A, Lee K. Interventions shown to aid executive function development in children 4 to 12 years old. Science 2011 Aug 19; 333(6045):959-64

[7] Blair C. Educating executive function. Wiley Interdiscip Rev Cogn Sci 2017 Jan; 8(1-2):10.1002/wcs.1403
