# Supplementary material for: Evaluating the Effectiveness of InsightApp for Anxiety, Valued Action, and Psychological Resilience: Longitudinal Randomized Controlled Trial
Source: JMIR Ment Health. 2025 Feb 4;12:e57201. doi: 10.2196/57201 (PMC11836588; doi:10.2196/57201)
Supplement: Multimedia Appendix 6 [file mental_v12i1e57201_app6.docx]

Multimedia Appendix 6 - Randomization and Blinding Process, and Power Analysis

## Randomization and Blinding Process

Participants were assigned to either the control or experimental group using **sequential allocation**, which was dynamically adjusted by the mobile application. Sequential allocation ensured an even distribution of participants between groups by assigning them one after another in a balanced manner. The randomization process was fully automated to prevent any potential human bias during participant assignment.

While the study was not **double-blinded**, participants were blinded to their group allocation to control for the **digital placebo effect**. Both groups were informed of the general purpose and expected benefits of the InsightApp and received credible interventions. The control group participated in metacognitive and cognitive exercises, which were similar to those used in executive function training programs and commercial brain training apps.

**Researchers had access to participants' Prolific IDs and group assignments** in order to send daily reminders about participation and the tasks to perform. This was necessary to ensure engagement and compliance with the study protocol. However, researchers did not have access to the data being collected during the experiment, maintaining the integrity of data collection.

## Power analysis

In our study, the primary objective was to ensure sufficient power for a longitudinal analysis, aimed at detecting small effects over time as participants interacted with the app during both the intervention and post-intervention phases. Using pilot data on struggle with emotion, we conducted Monte Carlo simulations to ensure 90% power for this specific longitudinal measure. Based on these results, we anticipated observing similar patterns for the other longitudinal outcomes. However, the study was not specifically optimized for detecting small effects in pre-post measures, and a larger sample size would be required to achieve adequate power for these analyses.

In the following section, we provide a detailed rationale behind the sample size selection and how it relates to the statistical power for both the longitudinal and pre-post analyses. While the longitudinal analysis was fully powered, the findings from the pre-post measures should be interpreted with caution, as a substantially larger sample would be necessary to reliably detect the magnitude of pre-post effects.

### Sample size calculation based on power analysis for longitudinal data analysis

To determine the required sample size for adequate statistical power, we conducted Monte Carlo power simulations using data from a pilot study. In these simulations, we treated the values observed in the pilot as population values (effect sizes, variances, etc.), which we then used to generate simulated samples. This approach allowed us to assess the power to detect significant effects across different sample sizes.

Using Mplus (Muthén & Muthén, 1998–2010), we specified a two-level random-effects model, simulating the effect of stress on emotional struggle over time. The model included both within-person variation in stress (str_cw) and emotional struggle at the within level, with fixed parameters taken from the pilot data.

For each sample size, we generated 1,000 simulated datasets. Each dataset was analyzed as if it were real, and the power of the model was determined by the proportion of samples in which the effect of interest (e.g., the impact of stress on struggle) was statistically significant. We aimed for a minimum power level of 80%, which is the standard threshold in research.

The power curve shows how power increases as the sample size increases. As illustrated in Figure S1, a sample size of 116 participants achieves 80% power, 132 participants achieve 85% power, and 162 participants reach 90% power. This analysis confirmed that our planned sample size was sufficient to ensure the study had the statistical power necessary to detect meaningful effects.

To account for potential participant dropout, we estimated a 40% dropout rate, which adjusted our required sample size to approximately 226 participants. This estimation ensured that even with attrition, we would still maintain sufficient statistical power to detect significant longitudinal effects.
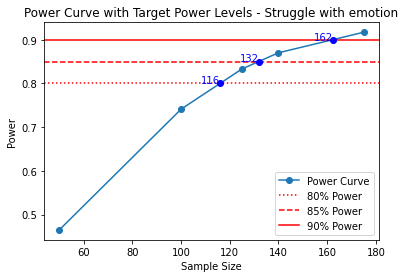


Figure S1: Power Curve with Target Power Levels – Struggle with Emotion. The power curve shows the relationship between sample size and statistical power for detecting significant effects of within-person variations in stress on emotional struggle. Power thresholds of 80%, 85%, and 90% are indicated by the red lines. The analysis shows that a sample size of 116 participants achieves 80% power, 132 participants reach 85% power, and 162 participants achieve 90% power.

### Pre-post power analysis

While the study was sufficiently powered for the longitudinal analysis, the pretest-posttest analysis had limitations in terms of power, particularly for detecting small effect sizes. However, it was well-powered for detecting medium and large effects.

The power analysis presented here demonstrates that, with a total sample size of 228 participants, the study was underpowered to detect small effects on the self-report measures administered in the post-test block and at follow-up. Specifically, for a small effect size (f=0.10), with α=0.05 and 1 covariate, the computed power was only 32.4% (F=3.88, numerator degrees of freedom = 1, denominator degrees of freedom = 225). This is far below the conventional 80% threshold, indicating that the study was not sufficiently powered to detect small changes.

In contrast, for medium effect sizes (f=0.25), the post hoc power analysis indicated that the sample size of 228 participants was well-powered. With the same model parameters, the computed power to detect a medium effect was 96.4% (critical F=3.88), which exceeds the 80% threshold. This shows that the study had a high probability of detecting medium-sized effects in the psychological measures.

In summary, while the study was well-powered for detecting medium effects, its ability to detect small effects was limited. This should be considered when interpreting the pretest-posttest results, as smaller effects may have gone undetected due to insufficient power. Given that we likely expected a smaller effect size, a larger sample size would have been required to achieve adequate power for detecting these smaller changes in the psychological measures.

## References

[1] Muthén LK, Muthén BO. Mplus User’s Guide. 6th ed. Los Angeles, CA: Muthén & Muthén; 1998-2010.
