# Supplementary material for: Evaluating the Effectiveness of InsightApp for Anxiety, Valued Action, and Psychological Resilience: Longitudinal Randomized Controlled Trial
Source: JMIR Ment Health. 2025 Feb 4;12:e57201. doi: 10.2196/57201 (PMC11836588; doi:10.2196/57201)
Supplement: Multimedia Appendix 7 [file mental_v12i1e57201_app7.docx]

Multimedia Appendix 7 - Baseline Comparison Between Groups

In Table S1, we present the statistical comparisons between the control and experimental groups for both demographic variables and baseline psychological scales. We conducted a series of statistical tests to assess whether any significant differences existed between the groups before the intervention. For categorical demographic variables, such as sex, ethnicity, country of residence, and employment status, we used Chi-square tests to evaluate group differences. For continuous variables, we first assessed normality using the Shapiro-Wilk test. Based on this assessment, we applied Mann-Whitney U tests to non-normally distributed variables like age.

We followed the same approach for the psychological scales. The t-test was used for BAFThoughts, the only scale with a normal distribution, while Mann-Whitney U tests were applied to all other scales, which were non-normally distributed. None of the comparisons revealed statistically significant differences between the groups, indicating that the control and experimental groups were comparable at baseline in terms of both demographic and psychological characteristics.

|  | Shapiro-test | Test | U-statistic | T-statistic | Chi^2 | DoF | *P*-value |
| --- | --- | --- | --- | --- | --- | --- | --- |
| Demographics |  |  |  |  |  |  |  |
| Age | non-normal | Mann-Whitney U | 4483.50 | - | - | - | 0.37 |
| Sex | - | Chi-square | - | - | 0.00 | 1 | 1.00 |
| Ethnicity | - | Chi-square | - | - | 3.96 | 4 | 0.41 |
| Country of Recidence | - | Chi-square | - | - | 0.55 | 1 | 0.46 |
| Employment status | - | Chi-square | - | - | 1.38 | 2 | 0.50 |
| Psychological scales |  |  |  |  |  |  |  |
| Mental well-being |  |  |  |  |  |  |  |
| BIThriving | non-normal | Mann-Whitney U | 5036.00 | - | - | - | 0.64 |
| SCompassion | non-normal | Mann-Whitney U | 4598.50 | - | - | - | 0.53 |
| PFlexibility | non-normal | Mann-Whitney U | 4672.50 | - | - | - | 0.66 |
| Mental health |  |  |  |  | - | - |  |
| ASensitivity | non-normal | Mann-Whitney U | 4985.00 | - | - | - | 0.74 |
| HARaiting | non-normal | Mann-Whitney U | 4434.00 | - | - | - | 0.30 |
| BAFThoughts | normal | T-test | - | 0.02 | - | 193.19 | 0.99 |
| Neuroticism | non-normal | Mann-Whitney U | 4593.00 |  | - | - | 0.52 |

**Table S1.** Statistical comparisons between the control and experimental groups for demographic variables and baseline psychological scales. We used Mann-Whitney U and t-tests for continuous variables based on their distribution, and Chi-square tests for categorical variables. None of the comparisons were statistically significant, confirming baseline equivalence between the groups.
