# Supplementary material for: Evaluating the Effectiveness of InsightApp for Anxiety, Valued Action, and Psychological Resilience: Longitudinal Randomized Controlled Trial
Source: JMIR Ment Health. 2025 Feb 4;12:e57201. doi: 10.2196/57201 (PMC11836588; doi:10.2196/57201)
Supplement: Multimedia Appendix 8 [file mental_v12i1e57201_app8.docx]

Multimedia Appendix 8 - Dropout, Compliance, Engagement, and Missing Data and Sparseness

## Dropout, Compliance, and Engagement

### Dropout

In this study, dropout was defined as participants who stopped participating at any point during the study and did not complete the offboarding measures, thereby leaving the study. Dropout rates were monitored across the different stages of the study in both the control and experimental conditions. Table S1 summarizes the number of dropouts at each stage:

Table S1. Number of participants who dropped out of the study by condition and stage

| Condition | Stage 1 | Stage 2 | Total per condition |
| --- | --- | --- | --- |
| Control | 10 | 2 | 12 |
| Experimental | 13 | 4 | 17 |

The retention rate, calculated as the percentage of participants who remained in the study until its conclusion after removing users who discontinued due to technical problems or failed attentional checks from stage 1 onwards (control = 4 and experimental = 0), was 89.3% (100 out of 112) in the control condition and 85.3% (99 out of 116) in the experimental condition. The total dropout rate, which accounts for participants who disengaged at any stage, was 10.7% (12 out of 112) in the control condition and 14.7% (17 out of 116) in the experimental condition. These figures indicate that while there was some attrition throughout the study, a significant portion of participants in both conditions remained engaged until the end.

The retention rate is calculated as the percentage of participants who stayed in the study until its conclusion. This calculation excludes those who discontinued due to technical problems or failed attentional checks from stage 1 onwards (control = 3 and 1, and experimental = 0). In the control condition, the retention rate was 89.3% (100 out of 112). In the experimental condition, the retention rate was 85.3% (99 out of 116). The total dropout rate per condition, which includes participants who disengaged at any stage, was 10.7% (12 out of 112) in the control condition and 14.7% (17 out of 116) in the experimental condition. These figures show that although there was some attrition throughout the study, a significant portion of participants in both conditions remained engaged until the end.

Stage 0 is not included in the dropout analysis comparing the control and experimental conditions, as all participants received the same intervention during this phase. However, it is noteworthy that 6 participants dropped out during Stage 0. Across the entire study, 29 out of 228 participants dropped out, which represents a dropout rate of approximately 12.7%. This dropout rate is substantially lower than the expected 40% drop, indicating higher-than-anticipated retention among participants.

### Compliance

In this study, compliance refers to the degree to which participants adhered to the study protocol by consistently engaging with the app and completing the required tasks across different stages. Specifically, in Stage 0, which lasted 4 days, participants needed to submit at least two evening reports. In Stage 1, spanning 7 days, participants were required to complete both the morning practice and the evening report on at least three days. Finally, in Stage 2, also 7 days long, participants needed to submit at least three evening reports. Meeting these criteria was essential for participants to be considered compliant, ensuring their continued participation in the study.

None of the participants who completed the study were excluded due to non-compliance. This indicates that all users in both the control and experimental conditions adhered to the participation requirements, resulting in 100% compliance in both groups.

### Engagement

In this study, engagement was assessed based on the active participation of users who completed the study. We specifically tracked two key metrics: the number of evening reports completed and the number of morning practices, with the latter being relevant only during Stage 1. Table S2 provides a summary of the average number of reports and practices completed by participants in each condition (control and experimental) across all stages.

Table S2. Average Number of Reports and Practices Completed by Stage and Condition

| Stage | Condition | Reports  Average (%) | Practices Average(%) |
| --- | --- | --- | --- |
| 0 | Not different | 3.92 (98%) | No practice required |
| 1 | Control | 6.9 (99%) | 6.71 (95%) |
| 1 | Experimental | 6.88 (98%) | 6.55 (93%) |
| 2 | Control | 6.51 (93%) | No practice required |
| 2 | Experimental | 6.42 (91%) | No practice required |

As shown in Table S2, the average completion rates for reports were consistently high across all stages and conditions. Participants in the control condition slightly outperformed those in the experimental condition in terms of report submission, with a minimal difference of about 1 to 2%. The engagement with morning practices during Stage 1 also demonstrated strong adherence, with control participants completing an average of 95% of the required practices, compared to 93% in the experimental group.

These findings reflect a high level of engagement among participants who completed the study. Both groups adhered closely to the daily task requirements, and the minor differences in task completion between the conditions suggest that participants across both conditions were similarly engaged throughout the study.

## Missing Data

In this study, missing data were primarily handled using PROC MIXED, which assumes that data are Missing at Random (MAR). To validate this assumption, we conducted both logistic regression and correlation analyses to evaluate the relationship between missingness and key observed variables (condition, stage, and day). It is important to note that missing data were minimal in this study. Out of a total of 3,403 expected reports across participants and days, only 143 were missing, representing a small fraction of the overall data. As detailed in the engagement section, completion rates for the reports were very high across all stages and conditions, with an average user engagement of 95.8%. The minimal amount of missing data further supports the robustness of our findings, as the low incidence of missingness reduces the likelihood of biasing the results.

### Logistic Regression Analysis

The logistic regression analysis aimed to predict the likelihood of missing reports based on the observed variables. The model revealed a low Pseudo R-squared value of 0.06134, indicating that the observed variables had limited explanatory power in predicting missingness. Although the variable 'day' (i.e., day of the study from 1 to 18) was statistically significant (p = 0.002), its effect size was small (odds ratio = 1.15). This suggests that while the timing within the study had some influence on missingness, it was not substantial enough to undermine the MAR assumption. Therefore, the missingness in the data seems to be weakly related to the observed data. However, it does not appear to be indicative of a Missing Not at Random (MNAR) mechanism. For variables that could plausibly confound the conclusions, the missing data appear to be missing at random. The day of the study is not systematically related to these potential confounding variables. Additionally, the day of the study does not differ between participants for whom the interventions were effective and those for whom they were not (condition), suggesting that the timing of missing data is unlikely to introduce bias into the outcomes. Table S3 summarizes the results of the logistic regression.

Table S3. Results for logistic regression

| **Logit Regression Results** | | | | | | | |
| --- | --- | --- | --- | --- | --- | --- | --- |
| Metric | | Value | | Metric | | Value | |
| Dependent Variable | | missing_report | | Df Residuals | | 3542 | |
| Model | | Logit | | Df Model | | 3 | |
| Method | | MLE | | Pseudo R-squ. | | 0.06134 | |
| Converge | | True | | Log-Likelihood | | -562.46 | |
| Covariance Type | | nonrobust | | LL-Null | | -599.21 | |
| No. Observations | | 3546 | | LLR p-value | | 7.563e-16 | |
|  | | **Coef** | **Std Err** | **z** | **P>\|z\|** | **[0.025** | **0.975]** |
| **const** | | -4.9566 | 0.282 | -17.575 | 0.000 | -5.509 | -4.404 |
| **condition** | | 0.1151 | 0.173 | 0.666 | 0.505 | -0.223 | 0.454 |
| **stage** | | 0.1161 | 0.330 | 0.351 | 0.725 | -0.532 | 0.764 |
| **day** | | 0.1385 | 0.045 | 3.095 | 0.002 | 0.051 | 0.226 |

### Correlation Analysis

We further examined the relationship between missingness and the key study variables (stage, day, and condition) using correlation analysis. The results showed slight correlations between the missingness of reports and the variables 'stage' (correlation = 0.13) and 'day' (correlation = 0.14). These correlations are very small, indicating that the influence of these variables on missingness is negligible. This finding aligns with the MAR assumption, suggesting that the missing data is likely related to observed factors but not in a way that would lead to MNAR. The detailed correlation values for each variable are presented in Figure S1.


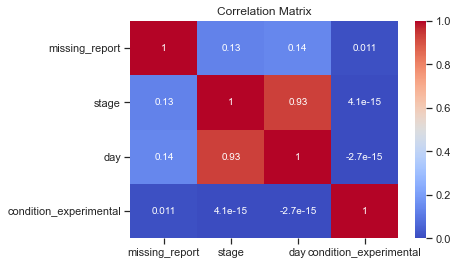


**Figure S1.** Correlation matrix of key variables showing the relationships between missing_report, stage, day, and condition_experimental. The matrix indicates that missing_report has a slight positive correlation with both stage (0.126) and day (0.140), though these correlations are relatively weak. The variable condition_experimental shows minimal correlation with other variables, highlighting its limited impact on missingness.

Based on the results of the logistic regression and correlation analyses, we find that the missingness in the data aligns with the Missing at Random (MAR) assumption. Given the significant but slight effect of 'day' on missingness, using MAR-based methods in our analysis is appropriate and ensures robust and reliable findings. The overall pattern of missingness does not suggest that the data is Missing Not at Random (MNAR).

## Data Sparseness

We further analyzed data sparsity across the study to assess the extent and potential impact of missing data. Overall, the level of sparsity was low, with only 4.03% of data missing (143 out of 3546 data points). This minimal amount of missing data suggests it is unlikely to introduce bias or significantly affect the robustness of the analysis, ensuring the results remain valid.

Missing data increased toward the end of the study, particularly in Stage 2, which had the highest proportion of missing data at 7.69%, compared to Stages 0 (1.90%) and 1 (1.60%). The highest levels of missing data were observed in the last two days of the study, with 10.66% missing on day 17 and 9.14% on day 18. This trend likely reflects participants achieving the required 4 out of 7 days of compliance and subsequently disengaging, no longer completing reports as the study approached its conclusion.

Figure S2 shows data sparsity, with participants displayed along the x-axis and the days of the study along the y-axis. Purple bars indicate missing evening surveys, while yellow bars represent completed surveys. This figure highlights patterns of disengagement, offering valuable insights for refining participant retention strategies in future research designs.


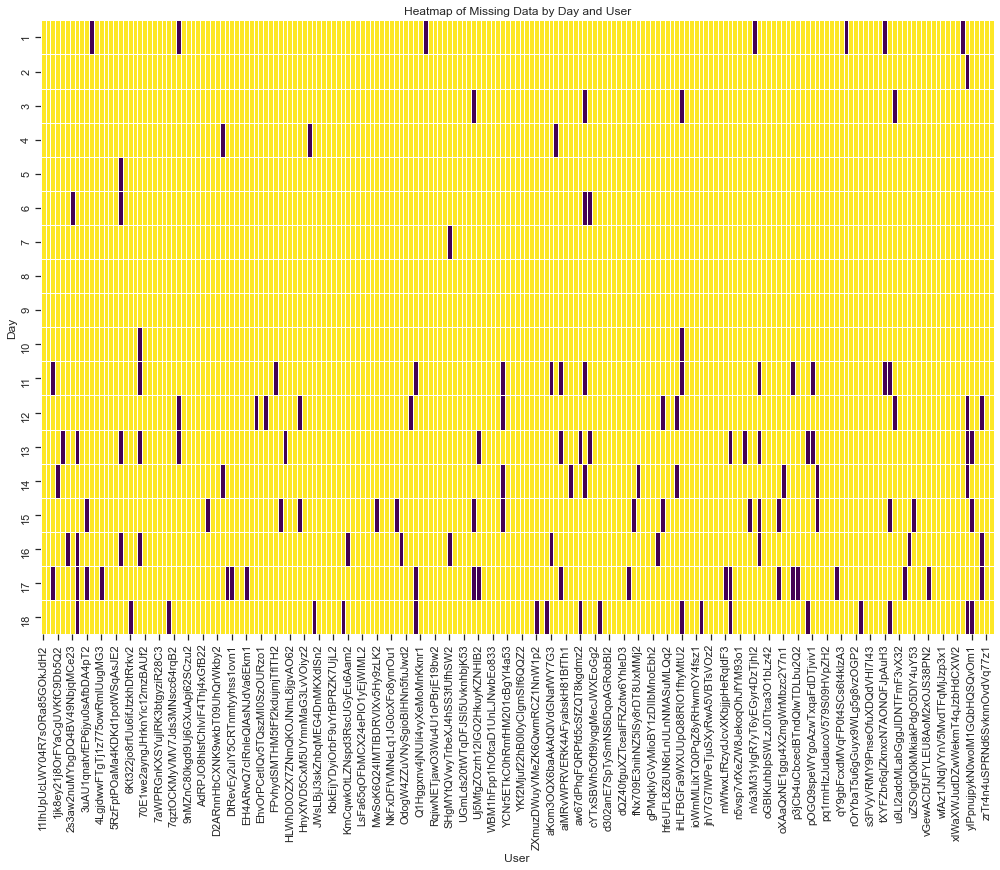


**Figure S2.** Data sparsity across the study period. The x-axis represents individual participants, and the y-axis shows each day of the intervention. Purple bars indicate missing evening surveys, while yellow bars represent completed surveys. Notable increases in missing data are observed towards the final stage and days of the study.
