# Supplementary material for: Evaluating the Effectiveness of InsightApp for Anxiety, Valued Action, and Psychological Resilience: Longitudinal Randomized Controlled Trial
Source: JMIR Ment Health. 2025 Feb 4;12:e57201. doi: 10.2196/57201 (PMC11836588; doi:10.2196/57201)
Supplement: Multimedia Appendix 9 [file mental_v12i1e57201_app9.docx]

Multimedia Appendix 9 - Baseline Characteristics and Pre-Post and Follow-up Assessments of Psychological Measures

## Baseline Characteristics

Table S1 provides the descriptive statistics for the psychological scales at baseline, summarizing the control and experimental groups before the intervention. The scales include measures of mental well-being (BIThriving, SCompassion, PFlexibility) and mental health (ASensitivity, HARaiting, BAFThoughts, Neuroticism). For each group, we report the mean, standard deviation (Std), median, and interquartile range (IQR), providing a detailed overview of participants' psychological states before the start of the experiment.

| **Scale** | **Condition** | **Mean** | **Std** | **Median** | **IQR** |
| --- | --- | --- | --- | --- | --- |
| BIThriving | control | 34.34 | 8.33 | 35 | 10.25 |
|  | experimental | 33.81 | 7.74 | 35 | 12 |
| SCompassion | control | 2.72 | 0.72 | 2.67 | 1.1 |
|  | experimental | 2.76 | 0.69 | 2.75 | 1 |
| PFlexibility | control | 26.17 | 11.41 | 25.5 | 18.25 |
|  | experimental | 26.71 | 10.8 | 25 | 15 |
| ASensitivity | control | 2.58 | 0.91 | 2.5 | 1.44 |
|  | experimental | 2.51 | 0.81 | 2.44 | 1.38 |
| HARaiting | control | 2.08 | 0.68 | 2.04 | 0.95 |
|  | experimental | 2.2 | 0.71 | 2 | 1.07 |
| BAFThoughts | control | 4.19 | 1.29 | 4.34 | 1.98 |
|  | experimental | 4.18 | 1.13 | 4.06 | 1.81 |
| Neuroticism | control | 6.47 | 2.44 | 7 | 5 |
|  | experimental | 6.7 | 2.4 | 7 | 4 |

**Table S1.** Descriptive statistics for psychological scales at baseline, including mental well-being and mental health. Data includes mean, standard deviation (Std), median, and interquartile range (IQR) for both control and experimental groups before the intervention.

## Anxiety Sample Characteristics

In this section, we analyze the baseline descriptive statistics for the mental health scales and examine the distribution of baseline anxiety among participants in both conditions. Although the study sample was drawn from a general, non-clinical population, the baseline anxiety measures reveal that participants exhibited a broad range of anxiety levels.

Anxiety Sensitivity scores ranged from 1.13 to 4.69 on a scale from 1 to 5, with an average score of 2.6 and a standard deviation of 0.86. This variation indicates that the participants exhibited a wide spectrum of sensitivity to anxiety, from very low to nearly the maximum possible score. Hamilton Anxiety Rating Scale revealed scores from 1.0 to 4.36, on a scale from 1 to 5. The mean score of 2.14 and a standard deviation of 0.69 suggest a predominantly moderate level of anxiety symptoms among the participants, with few reaching the upper limits of the scale. Cognitive Fusion with Anxiety scores were observed from 1.5 to the full-scale maximum of 7.0. The mean of 4.19 and a standard deviation of 1.21 reflect a broad engagement with anxious thoughts, indicating variability in how participants' thoughts might influence their emotional responses. Neuroticism scores presented the widest range of scores, from 2.0 to 10.0 on a scale from 1 to 10, with an average score of 6.58 and a substantial standard deviation of 2.42. This range highlights significant differences in trait neuroticism, suggesting diverse predispositions to stress and emotional instability across the sample.

Figure S1 presents the distribution of baseline anxiety scores for Anxiety Sensitivity, the Hamilton Anxiety Rating Scale, Cognitive Fusion with Anxiety, and Neuroticism, taking into account the maximum scores possible on each scale. Participants were categorized into "low," "moderate," and "high" anxiety levels based on the 25th and 75th percentiles of their scores. For Anxiety Sensitivity, out of 197 participants, 45 (22.84%) were classified as low, 100 (50.76%) as moderate, and 52 (26.40%) as high. For the Hamilton Anxiety Rating Scale, out of 197 participants, 48 (24.37%) had low scores, 98 (49.75%) were moderate, and 51 (25.89%) were high. For Cognitive Fusion with Anxiety, out of 197 participants, 48 (24.37%) were classified as low, 98 (49.75%) as moderate, and 51 (25.89%) as high. For Neuroticism, out of 197 participants, 20 (10.15%) were in the low category, 123 (62.44%) were moderate, and 54 (27.41%) were high. These results demonstrate a broad range of anxiety levels within the sample, with a substantial proportion of participants experiencing moderate to high anxiety traits.


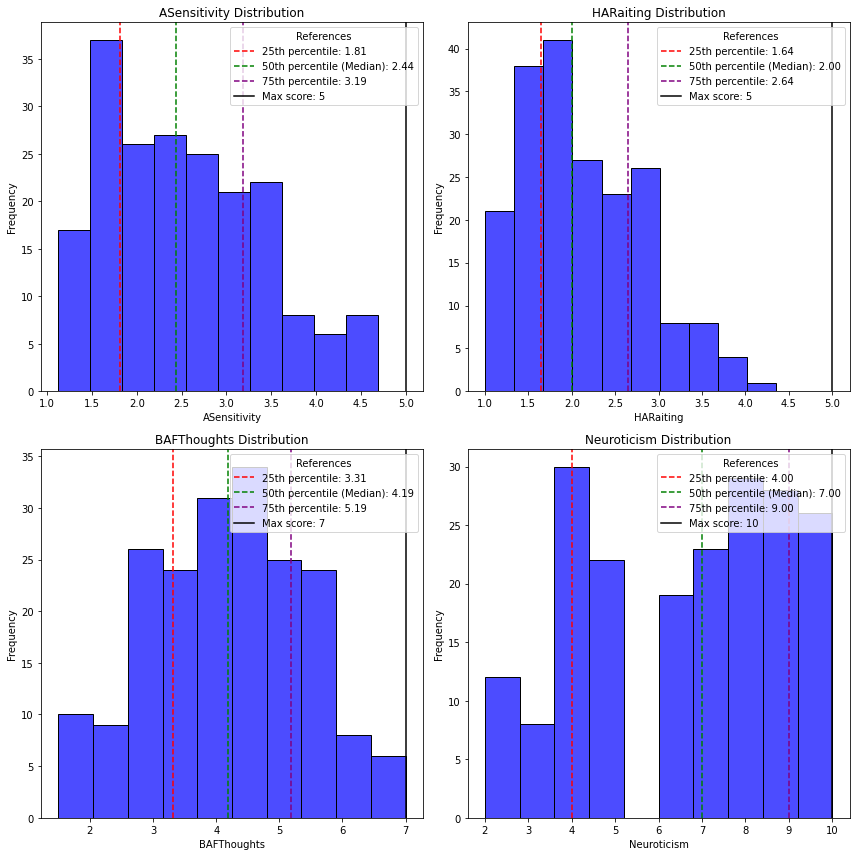


**Figure S1**. Distribution of Anxiety-Related Measures in the Baseline Sample Histograms illustrate the distribution of baseline scores for Anxiety Sensitivity, Hamilton Anxiety Rating Scale (HARaiting), Cognitive Fusion (BAFThoughts), and Neuroticism. Vertical lines represent the 25th percentile (red), 50th percentile (green), and 75th percentile (purple). A black line indicates the maximum possible score for each scale. Participants were categorized into "low," "moderate," and "high" based on the 25th and 75th percentiles. The y-axis shows the number of participants within each score range, while the x-axis displays the respective scores for each scale.

In addition to the baseline anxiety measures, participants reported varying levels of emotional struggle and anxiety intensity related to these situations. Figure S2 shows the distributions for the situation and emotion regularity for both the control and experimental conditions. The graph displays how often participants in each group encountered anxiety-provoking situations (situation regularity) and how frequently they experienced emotional responses to these situations (emotion regularity). The comparison highlights that the distribution of regularity in encountering anxiety-triggering situations and emotional reactions was relatively similar between the two groups, further illustrating the consistency of the study population's exposure to stressful events across both conditions.

The distribution of anxiety intensity and struggle showed that participants regularly faced anxiety-provoking situations during the study, with notable variability in their emotional responses. Specifically, 134 (134/197, 68.0%) participants reported encountering anxiety-triggering situations daily, 21 (21/197, 10.7%) participants experienced them every second day, and 31 (31/197, 15.7%) encountered them weekly. Only 7 (7/197, 3.6%) participants faced these situations monthly, while 4 (4/197, 2.0%) were unsure or could not specify how frequently they encountered such situations. Similarly, when reporting on their general situation regularity, 159 (159/197, 80.7%) participants indicated they experienced these situations daily, 12 (12/197, 6.1%) reported every second day, 18 (18/197, 9.1%) weekly, and 7 (7/197, 3.6%) monthly, with just 1 (1/197, 0.5%) stating they did not often encounter such situations.


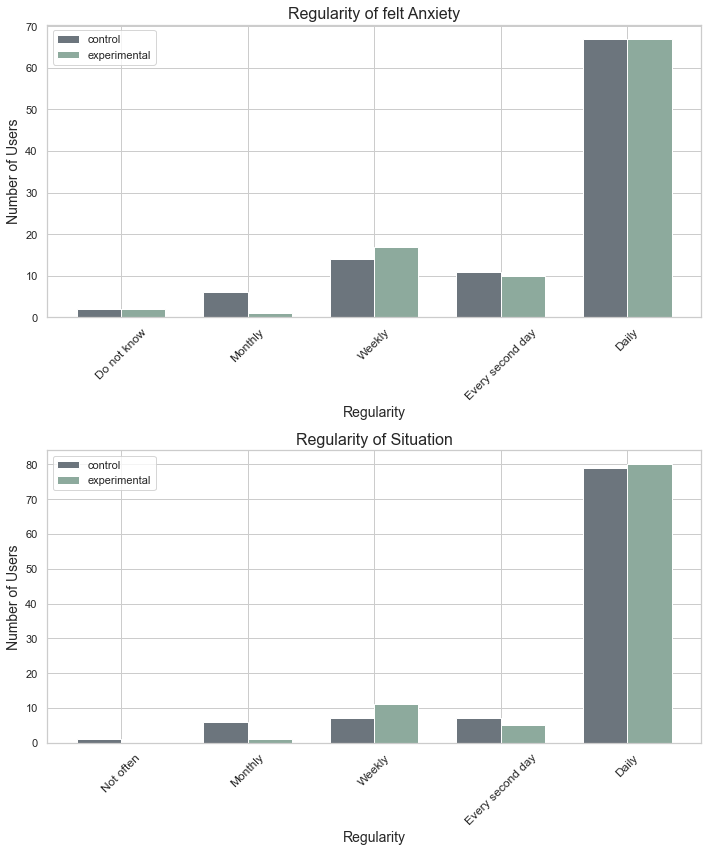


**Figure S2.** Distribution of Users by Emotion Regularity and Situation Regularity across Control and Experimental Conditions. The top plot shows the number of users who reported encountering emotional triggers with different regularities (e.g., daily, weekly) in both the control and experimental conditions. The bottom plot displays how often users encountered anxiety-provoking situations, with similar categorizations across control and experimental groups. Larger text is used for readability. Both plots highlight the differences in frequency and distribution between the two conditions.

## Pre-Post and Follow-up Psychological Surveys

In this section, we present the descriptive statistics for the psychological scales measured at three-time points: pre-intervention (baseline), post-intervention, and follow-up. The data is divided into two categories: Mental Well-Being and Mental Health. For each scale, we provide the mean, standard deviation (Std), median, and interquartile range (IQR) across the control and experimental groups.

The Mental Well-Being scales include BIThriving, SCompassion, and PFlexibility, while the Mental Health scales consist of ASensitivity, HARaiting, BAFThoughts, and Neuroticism. These statistics give a comprehensive overview of the psychological state of participants at each time point, helping to track changes over time within and between groups. Table S2 contains the descriptive statistics for mental well-being and mental health scales across the pre-intervention, post-intervention, and follow-up time points.

**Table S2.** Descriptive statistics for mental well-being and mental health scales across pre-intervention, post-intervention, and follow-up time points. The table includes data for both control and experimental groups, reporting mean, standard deviation (Std), median, and interquartile range (IQR) for each time point

| Mental Well-being |  | Pre | Pre | Pre | Pre | Post | Post | Post | Post | Follow | Follow | Follow | Follow |
| --- | --- | --- | --- | --- | --- | --- | --- | --- | --- | --- | --- | --- | --- |
| Scale | Condition | Mean | Std | Median | IQR | Mean | Std | Median | IQR | Mean | Std | Median | IQR |
| BIThriving | control | 34.34 | 8.33 | 35 | 10.25 | 34.7 | 8.57 | 35 | 10.25 | 35.44 | 8.87 | 36.5 | 10.5 |
| BIThriving | experimental | 33.81 | 7.74 | 35 | 12 | 34.62 | 7.92 | 36 | 11 | 35.45 | 7.95 | 37 | 10 |
| SCompassion | control | 2.72 | 0.72 | 2.67 | 1.1 | 2.87 | 0.68 | 2.71 | 1 | 2.89 | 0.72 | 2.88 | 1.02 |
| SCompassion | experimental | 2.76 | 0.69 | 2.75 | 1 | 2.87 | 0.61 | 2.83 | 0.92 | 2.96 | 0.66 | 3 | 0.75 |
| PFlexibility | control | 26.17 | 11.41 | 25.5 | 18.25 | 24.54 | 10.67 | 24 | 17 | 24.35 | 11.05 | 23.5 | 17.25 |
| PFlexibility | experimental | 26.71 | 10.8 | 25 | 15 | 25.41 | 9.44 | 25 | 12 | 24.93 | 9.71 | 26 | 15 |
| Mental health |  | Pre | Pre | Pre | Pre | Post | Post | Post | Post | Follow | Follow | Follow | Follow |
| Scale | Condition | Mean | Std | Median | IQR | Mean | Std | Median | IQR | Mean | Std | Median | IQR |
| ASensitivity | control | 2.58 | 0.91 | 2.5 | 1.44 | 2.48 | 0.88 | 2.31 | 1.33 | 2.28 | 0.84 | 2.12 | 1.19 |
| ASensitivity | experimental | 2.51 | 0.81 | 2.44 | 1.38 | 2.37 | 0.84 | 2.12 | 1.06 | 2.22 | 0.87 | 1.94 | 1.19 |
| HARaiting | control | 2.08 | 0.68 | 2.04 | 0.95 | 4.01 | 1.25 | 4.09 | 1.84 | 3.97 | 1.31 | 4.09 | 2.06 |
| HARaiting | experimental | 2.2 | 0.71 | 2 | 1.07 | 1.99 | 0.62 | 1.86 | 0.86 | 1.91 | 0.65 | 1.79 | 1 |
| BAFThoughts | control | 4.19 | 1.29 | 4.34 | 1.98 | 4.01 | 1.25 | 4.09 | 1.84 | 3.97 | 1.31 | 4.09 | 2.06 |
| BAFThoughts | experimental | 4.18 | 1.13 | 4.06 | 1.81 | 4.09 | 1.13 | 4.12 | 1.88 | 3.97 | 1.23 | 3.94 | 2 |
| Neuroticism | control | 6.47 | 2.44 | 7 | 5 | 6.33 | 2.45 | 6.5 | 4 | 6.06 | 2.41 | 6 | 4 |
| Neuroticism | experimental | 6.7 | 2.4 | 7 | 4 | 6.37 | 2.13 | 6 | 3 | 6.06 | 2.22 | 6 | 4 |
