# Supplementary material for: Evaluating the Effectiveness of InsightApp for Anxiety, Valued Action, and Psychological Resilience: Longitudinal Randomized Controlled Trial
Source: JMIR Ment Health. 2025 Feb 4;12:e57201. doi: 10.2196/57201 (PMC11836588; doi:10.2196/57201)
Supplement: Multimedia Appendix 10 [file mental_v12i1e57201_app10.docx]

Multimedia Appendix 10 - Results Tables for Intervention Period and Exploratory Analysis

## Results tables for intervention period

**Table S1.** Results for the intensity of participants’ anxiety during the intervention phase. Each row corresponds to a predictor in a mixed linear model, with the intensity of anxiety as the dependent variable.

| Solution for Fixed Effects | | | | | | | |
| --- | --- | --- | --- | --- | --- | --- | --- |
| Effect | Estimate | Standard  Error | DF^a^ | t Value | P value^b^ | 95% Confidence Interval | |
|  |  |  |  |  |  | Lower | Upper |
| Intercept | 32.1747 | 4.8441 | 191 | 6.64 | <.0001 | 22.6198 | 41.7296 |
| Intensity pre^c^ | 0.1476 | 0.04075 | 197 | 3.62 | 0.0004 | 0.06724 | 0.228 |
| Self-compassion pre | -2.5378 | 1.1399 | 197 | -2.23 | 0.0271 | -4.7856 | -0.2899 |
| Anxiety sensitivity pre | 1.2131 | 0.8151 | 197 | 1.49 | 0.1383 | -0.3944 | 2.8205 |
| Unrelated stressors | 0.2 | 0.1421 | 197 | 1.41 | 0.1608 | -0.08018 | 0.4802 |
| Condition | -2.0523 | 1.2639 | 197 | -1.62 | 0.106 | -4.5449 | 0.4402 |
| Between stress | 7.3718 | 0.6384 | 197 | 11.55 | <.0001 | 6.1129 | 8.6307 |
| Condition*between stress | 0.1296 | 0.6885 | 197 | 0.19 | 0.8509 | -1.2281 | 1.4873 |
| Within stress | 8.9975 | 0.3993 | 197 | 22.53 | <.0001 | 8.21 | 9.785 |
| Condition*within stress | -1.3543 | 0.5686 | 197 | -2.38 | 0.0091 | -2.4757 | -0.2329 |
| Time^d^ | -0.2389 | 0.1601 | 197 | -1.49 | 0.1372 | -0.55460 | 0.07678 |
| Solution for Random Effects | | | | | | | |
| Cov Parm | Subject | Estimate | Standard  Error | Z Value | P value | 95% Confidence Interval | |
|  |  |  |  |  |  | Lower | Upper |
| UN(1,1) | ID | 51.9816 | 8.0481 | 6.46 | <.0001 | 39.2171 | 72.2121 |
| UN(2,1) | ID | 8.9583 | 2.6165 | 3.42 | 0.0006 | 3.8301 | 14.0865 |
| UN(2,2) | ID | 6.6012 | 1.5438 | 4.28 | <.0001 | 4.378 | 11.0872 |
| SP(POW)^e^ | ID | 0.1027 | 0.04444 | 2.31 | 0.0208 | 0.01564 | 0.1898 |
| Residual |  | 108.6 | 5.6059 | 19.37 | <.0001 | 98.4005 | 120.48 |

^a^ As advised by Bolger and Laurenceau (2013), we adopted a conservative approach in determining the degrees of freedom, utilizing the number of subjects (N = 197) rather than the total number of observations.

^b^ All *P*-values reported in the analysis are two-tailed, except for the interaction of condition*within stress, for which one-tailed p-values are used in accordance with the pre-registered directional hypotheses.

^c^ *Intensity pre* refers to the average intensity of participants’ anxiety during the 4-day pre intervention period.

^d^ As advised by Bolger and Laurenceau (2013), the variable time was rescaled such that 0 corresponds to the middle of the intervention period. A 1-unit difference in the variable time represents the passage of one day.

^e^ Although the autocorrelation in the data is statistically significant, it is relatively small in magnitude, with a value of 0.1027. This indicates that there is a weak positive autocorrelation in the level-1 (within-subject) residuals.

**Table S2.** Results for the participants’ struggle with anxiety during the intervention phase. Each row corresponds to a predictor in a mixed linear model, with struggle with anxiety as the dependent variable.

| Solution for Fixed Effects | | | | | | | |
| --- | --- | --- | --- | --- | --- | --- | --- |
| Effect | Estimate | Standard  Error | DF^a^ | t Value | P value^b^ | 95% Confidence Interval | |
|  |  |  |  |  |  | Lower | Upper |
| Intercept | 29.8763 | 4.8796 | 191 | 6.12 | <.0001 | 20.2516 | 39.5011 |
| Struggle pre^c^ | 0.1169 | 0.04085 | 197 | 2.86 | 0.0047 | 0.03636 | 0.1975 |
| self-compassion pre | -3.2628 | 1.1436 | 197 | -2.85 | 0.0048 | -5.5181 | -1.0076 |
| anxiety sensitivity pre | 0.186 | 0.08723 | 197 | 2.13 | 0.0342 | 0.01396 | 0.358 |
| unrelated stressors | 1.1172 | 0.1494 | 197 | 7.48 | <.0001 | 0.8224 | 1.4119 |
| condition | -1.1766 | 1.3081 | 197 | -0.9 | 0.3695 | -3.7563 | 1.4031 |
| between stress | 6.4537 | 0.6344 | 197 | 10.17 | <.0001 | 5.2026 | 7.7047 |
| condition*between stress | -0.3174 | 0.7005 | 197 | -0.45 | 0.651 | -1.6989 | 1.0642 |
| within stress | 8.8614 | 0.401 | 197 | 22.1 | <.0001 | 8.0707 | 9.6522 |
| condition*within stress | -1.0713 | 0.5723 | 197 | -1.87 | 0.03135 | -2.1998 | 0.05731 |
| Time^d^ | -0.1121 | 0.1729 | 197 | -0.65 | 0.5178 | -0.4531 | 0.229 |
| Solution for Random Effects | | | | | | | |
| Cov Parm | Subject | Estimate | Standard  Error | Z Value | P value | 95% Confidence Interval | |
|  |  |  |  |  |  | Lower | Upper |
| UN(1,1) | ID | 54.3266 | 8.7889 | 6.18 | <.0001 | 40.509 | 76.6906 |
| UN(2,1) | ID | 12.2832 | 2.8308 | 4.34 | <.0001 | 6.735 | 17.8315 |
| UN(2,2) | ID | 6.1753 | 1.5393 | 4.01 | <.0001 | 3.9982 | 10.7836 |
| SP(POW)^e^ | ID | 0.1386 | 0.04424 | 3.13 | 0.0017 | 0.05187 | 0.2253 |
| Residual |  | 125.01 | 6.5993 | 18.94 | <.0001 | 113.02 | 139.02 |

^a^ As advised by Bolger and Laurenceau (2013), we adopted a conservative approach in determining the degrees of freedom, utilizing the number of subjects (N = 197) rather than the total number of observations.

^b^ All p-values reported in the analysis are two-tailed, except for the interaction of condition*within stress, for which one-tailed p-values are used in accordance with the pre-registered directional hypotheses.

^c^ *Struggle pre* refers to the average degree to which participants struggled with anxiety during the 4-days pre intervention period.

^d^ As advised by Bolger and Laurenceau (2013), the variable time was rescaled such that 0 corresponds to the middle of the intervention period. A 1-unit difference in the variable time represents the passage of one day.

^e^ Although the autocorrelation in the data is statistically significant, it is relatively small in magnitude, with a value of 0.044. This indicates that there is a weak positive autocorrelation in the level-1 (within-subject) residuals.

**Table S3**. Results for the participants’ enactment of their valued action during the intervention phase. Each row corresponds to a predictor in a mixed linear model, with valued intention enactment as the dependent variable.

| Solution for Fixed Effects | | | | | | | |
| --- | --- | --- | --- | --- | --- | --- | --- |
| Effect | Estimate | Standard  Error | DF^a^ | t Value | P value^b^ | 95% Confidence Interval | |
|  |  |  |  |  |  | Lower | Upper |
| Intercept | -7.6546 | 6.8849 | 191 | -1.11 | 0.2676 | -21.2348 | 5.9256 |
| Valued action pre^c^ | 0.7303 | 0.04078 | 197 | 17.91 | <.0001 | 0.6499 | 0.8107 |
| strategy 1 pre | 2.9923 | 1.2017 | 197 | 2.49 | 0.0136 | 0.6225 | 5.3622 |
| strategy 2 pre | 3.261 | 1.4478 | 197 | 2.25 | 0.0254 | 0.4059 | 6.1161 |
| Unrelated stressors | -0.06137 | 0.2537 | 197 | -0.24 | 0.8091 | -0.5617 | 0.439 |
| Condition | 7.0787 | 2.1927 | 197 | 3.23 | 0.0015 | 2.7545 | 11.4029 |
| Between stress | 0.5537 | 0.8938 | 197 | 0.62 | 0.5363 | -1.209 | 2.3163 |
| Condition*between stress | -0.8673 | 1.2421 | 197 | -0.7 | 0.4858 | -3.3168 | 1.5821 |
| Within stress | -2.8 | 0.742 | 197 | -3.77 | 0.0002 | -4.2633 | -1.3366 |
| Condition*within stress | 1.0485 | 1.0531 | 197 | 1 | 0.16035 | -1.0283 | 3.1253 |
| Time^d^ | 0.3632 | 0.2786 | 197 | 1.3 | 0.1938 | -0.1862 | 0.9127 |
| Solution for Random Effects | | | | | | | |
| Cov Parm | Subject | Estimate | Standard  Error | Z Value | P value | 95% Confidence Interval | |
|  |  |  |  |  |  | Lower | Upper |
| UN(1,1) | ID | 165.25 | 24.2368 | 6.82 | <.0001 | 126.41 | 225.31 |
| UN(2,1) | ID | -17.5442 | 8.1376 | -2.16 | 0.0311 | -33.4935 | -1.5949 |
| UN(2,2) | ID | 23.9965 | 5.5836 | 4.3 | <.0001 | 15.9446 | 40.184 |
| SP(POW)^e^ | ID | 0.02898 | 0.03878 | 0.75 | 0.4549 | -0.04702 | 0.105 |
| Residual |  | 346.79 | 16.8296 | 20.61 | <.0001 | 316.03 | 382.29 |

^a^ As advised by Bolger and Laurenceau (2013), we adopted a conservative approach in determining the degrees of freedom, utilizing the number of subjects (N = 197) rather than the total number of observations.

^b^ All p-values reported in the analysis are two-tailed, except for the interaction of condition*within stress, for which one-tailed p-values are used in accordance with the pre-registered directional hypotheses.

^c^ Since the effect of condition on valued action belongs to the set of exploratory hypotheses reported in the preregistration, we employed a Benjamini-Hochberg procedure to control the false discovery rate (FDR) at a 5% level. The p-value was found to be smaller than the Benjamini-Hochberg critical value (P=0.0015< 0.002), indicating a statistically significant result while controlling for the false discovery rate.

^c^ *Valued action pre* refers to the average amount of valued action enacted by participants’s during the 4-days pre intervention period.

^d^ As advised by Bolger and Laurenceau (2013), the variable time was rescaled such that 0 corresponds to the middle of the intervention period. A 1-unit difference in the variable time represents the passage of one day.

^e^ As shown by the magnitude (0.038) and p-value of the coefficient (P=0.45), there is no evidence of autocorrelation in the level-1 (within-subject) residuals.

## Exploratory Analyses: Impact of Anxiety-Related Variables on Intervention Effectiveness

We conducted exploratory analyses to determine whether the effectiveness of the intervention in reducing emotional struggle, the intensity of anxiety, and alignment with valued actions varied based on participants' baseline levels of anxiety-related traits, including Anxiety Sensitivity, Hamilton Anxiety Rating, Cognitive Fusion with Anxiety, and Neuroticism. After incorporating relevant interaction terms into our models (scale, scale*condition, scale*str_ce, and scale*str_cw*condition), we found that the three-way interactions between condition, within-person stress variation (str_cw), and baseline anxiety traits were not statistically significant, either during the intervention or in the post-intervention phase. Table S4 provides a detailed summary of both the three-way interactions (condition * str_cw * baseline trait) and two-way interactions (condition * baseline trait) for both the intervention and post-intervention periods.

As indicated in the table, none of the three-way interactions reached statistical significance (all p-values > 0.05), suggesting that baseline levels of anxiety traits did not significantly moderate the intervention's impact on stress reactivity. Similarly, the two-way interactions between condition and each baseline trait were not significant, indicating that the intervention's effectiveness was not dependent on initial anxiety levels alone. The table also includes estimates for emotion intensity, emotional struggle, and alignment with valued actions.

**Table S4**. Interaction Effects of Baseline Anxiety Traits on Intervention Outcomes During and Post-Intervention

| **Intervention period** | | | | | | |
| --- | --- | --- | --- | --- | --- | --- |
| **Outcome measure** | **Scale** | **Interaction term** | **Estimate** | **Standard**  **Error** | **t Value** | **Pr > \|t\|** |
| **Emotion Intensity** | **HARaiting** | condition*str_cw*scale | -0.00171 | 0.06397 | -0.03 | 0.9787 |
|  |  | condition*scale | -0.1045 | 0.166 | -0.63 | 0.5296 |
|  | **Neuroticism** | condition*str_cw*scale | -0.2032 | 0.2466 | -0.82 | 0.4109 |
|  |  | condition*scale | -0.847 | 0.6107 | -1.39 | 0.167 |
|  | **ASensitivity** | condition*str_cw*scale | -0.9714 | 0.6596 | -1.47 | 0.1424 |
|  |  | condition*scale | 1.2521 | 1.5838 | 0.79 | 0.4301 |
|  | **BAFThoughts** | condition*str_cw*scale | -0.4213 | 0.5079 | -0.83 | 0.4078 |
|  |  | condition*scale | 0.2622 | 1.1695 | 0.22 | 0.8229 |
| **Emotion Struggle** | **HARaiting** | condition*str_cw*scale | 0.07484 | 0.0633 | 1.18 | 0.2385 |
|  |  | condition*scale | -0.05735 | 0.1734 | -0.33 | 0.7412 |
|  | **Neuroticism** | condition*str_cw*scale | 0.3976 | 0.2463 | 1.61 | 0.1081 |
|  |  | condition*scale | 0.04704 | 0.6347 | 0.07 | 0.941 |
|  | **ASensitivity** | condition*str_cw*scale | -0.111 | 0.6697 | -0.17 | 0.8685 |
|  |  | condition*scale | 0.5965 | 1.6536 | 0.36 | 0.7187 |
|  | **BAFThoughts** | condition*str_cw*scale | 0.0306 | 0.5031 | 0.06 | 0.9516 |
|  |  | condition*scale | 0.6236 | 1.2261 | 0.51 | 0.6116 |
| **Valued Action** | **HARaiting** | condition*str_cw*scale | -0.02408 | 0.1179 | -0.2 | 0.8384 |
|  |  | condition*scale | -0.01413 | 0.2952 | -0.05 | 0.9619 |
|  | **Neuroticism** | condition*str_cw*scale | -0.9008 | 0.4621 | -1.95 | 0.0527 |
|  |  | condition*scale | -0.29 | 1.0835 | -0.27 | 0.7892 |
|  | **ASensitivity** | condition*str_cw*scale | -1.0406 | 1.2383 | -0.84 | 0.4018 |
|  |  | condition*scale | -3.8547 | 2.7822 | -1.39 | 0.1675 |
|  | **BAFThoughts** | condition*str_cw*scale | -0.9008 | 0.4621 | -1.95 | 0.0527 |
|  |  | condition*scale | -0.29 | 1.0835 | -0.27 | 0.7892 |
| **Post- intervention period** | | | | | | |
| **Outcome measure** | **Scale** | **Interaction term** | **Estimate** | **Standard**  **Error** | **t Value** | **Pr > \|t\|** |
| **Emotion Struggle** | **HARaiting** | condition*str_cw*scale | 0.04077 | 0.05547 | 0.73 | 0.4633 |
|  |  | condition*scale | 0.1109 | 0.1564 | 0.71 | 0.4792 |
|  | **Neuroticism** | condition*str_cw*scale | 0.2933 | 0.2221 | 1.32 | 0.1881 |
|  |  | condition*scale | 0.9104 | 0.5836 | 1.56 | 0.1204 |
|  | **ASensitivity** | condition*str_cw*scale | 0.2926 | 0.6024 | 0.49 | 0.6278 |
|  |  | condition*scale | 1.8477 | 1.4858 | 1.24 | 0.2151 |
|  | **BAFThoughts** | condition*str_cw*scale | -0.08889 | 0.4431 | -0.2 | 0.8412 |
|  |  | condition*scale | 1.5253 | 1.0597 | 1.44 | 0.1516 |

## References

Bolger N, Davis A, Rafaeli E. Diary methods: capturing life as it is lived. Annu Rev Psychol 2003; 54(1):579-616
