# Supplementary material for: Evaluating the Effectiveness of InsightApp for Anxiety, Valued Action, and Psychological Resilience: Longitudinal Randomized Controlled Trial
Source: JMIR Ment Health. 2025 Feb 4;12:e57201. doi: 10.2196/57201 (PMC11836588; doi:10.2196/57201)
Supplement: Multimedia Appendix 11 [file mental_v12i1e57201_app11.docx]

Multimedia Appendix 11 - Results for the Post-Intervention Period

We employed mixed-effects linear regressions to examine the effect of stress on participants' daily scores of anxiety intensity, struggle with anxiety, and intention enactment during the 7-day post-intervention period. The spaghetti plots in Figure S1 illustrate the qualitative relationships between within-person variation in stress and daily scores for each outcome measure. Visually, the regression lines for struggle with anxiety (middle panel B) appear to be less steep for the treatment group than for the control group. This suggests that individuals in the treatment group might have exhibited lower reactivity to stress concerning their struggle with anxiety after the app functionalities were removed. However, this effect does not seem to be the case for the intensity of anxiety (lower panel A) and for intention enactment (lower panel C).

In the following, we present the results for each outcome measure separately. First, we report the main effect of condition. Subsequently, to understand participants' reactivity to stress during the intervention period, we analyze how the outcome variable varies as a result of a unit of increase within-participant stress for each condition. Finally, we report the relative difference in reactivity to stress between groups.

Intensity of anxiety: The regression analyses summarized in Table S1 indicate that there was no significant main effect of condition on the average intensity of participants' anxiety (t₁₉₇=-0.38; *P*=.7; 95% CI -2.95-1.99) during the post-intervention period. In the control group, we found that the intensity of participants' anxiety increased by 7.77 units (on a scale from 0 to 100) per unit of within-person variation in stress (t₁₉₇=22.13; *P*=<.001; 95% CI 7.08-8.46). In the treatment group, the intensity of participants' anxiety increased by 7 units per unit of within-person variation in stress (t₁₉₇=19.62; *P*<.001; 95% CI 6.29-7.7). The interaction between stress and condition had no significant effect on the intensity of participants anxiety (t₁₉₇ = -1.57; P =.06; 95% CI -1.75 to 0.2).

Struggle with anxiety: The regression analyses summarized in Table S2 indicate that there was no significant main effect of condition on the degree to which participants struggled with anxiety (t₁₉₇=-1.38; *P*=.17; 95% CI -4.07-0.72) during the post-intervention period. In the control group, we found that the strength of participants’ struggle with anxiety increased by 7.93 units of struggle per unit of within-person variation in stress (t₁₉₇=22.17; *P*=<.001; 95% CI 7.23-8.64). In the treatment group, the strength of participants’ struggle with anxiety increased by 6.51 units of struggle per unit of within-person variation in stress (t₁₉₇=17.87; *P*<.001; 95% CI 5.79-7.22). This indicates that the degree to which participants struggled with anxiety in response to one unit of stress continued to be significantly lower in the experimental group by 1.43 units (t₁₉₇=-2.84; *P*<.01; 95% CI -2.42--0.44) relative to the control group. In other words, for each unit of stress, the experimental group’s amount of struggle with anxiety increased 17.99% less than the control group, once the app functionalities were taken away.

Valued intention enactment: The regression analyses summarized in Table S3 indicate that there was no significant main effect of condition on the degree to which people enacted their valued intentions (t₁₉₇=-1.93; *P*=0.6; 95% CI -0.1-9.06) during the post-intervention period. In the control group, the degree to which participants enacted the valued intentions decreased by 2.94 units per unit of within-person variation in stress (t₁₉₇=-3.49; *P*=<.001; 95% CI -4.61--1.28). In the treatment group, the degree to which participants enacted the valued intention in the treatment condition decreased by 1.32 units (t₁₉₇=-1.55; *P*=.12; 95% CI -3.01-0.37) per unit of within-person variation in stress. The interaction between stress and condition had no significant effect on intention enactment (t₁₉₇=1.36; P =.09; 95% CI -0.73 to 3.98).

Finally, the autocorrelation of the data was statistically significant for the intensity of participants’ anxiety (SP(POW)=0.12, z₁₉₇=2.81;*P*=0.01, 95% CI 0.034-0.19), how much they struggled with anxiety (SP(POW)=0.11, z₁₉₇=2.82; *P*<0.01, 95% CI 0.034-0.19), and how much they enacted their valued intentions (SP(POW)=0.18, z₁₉₇=4.03; *P*<0.001, 95% CI 0.092-0.267). However, the magnitude of the autocorrelation coefficients indicates only a weak positive autocorrelation in the level-1 (within-subject) residuals.


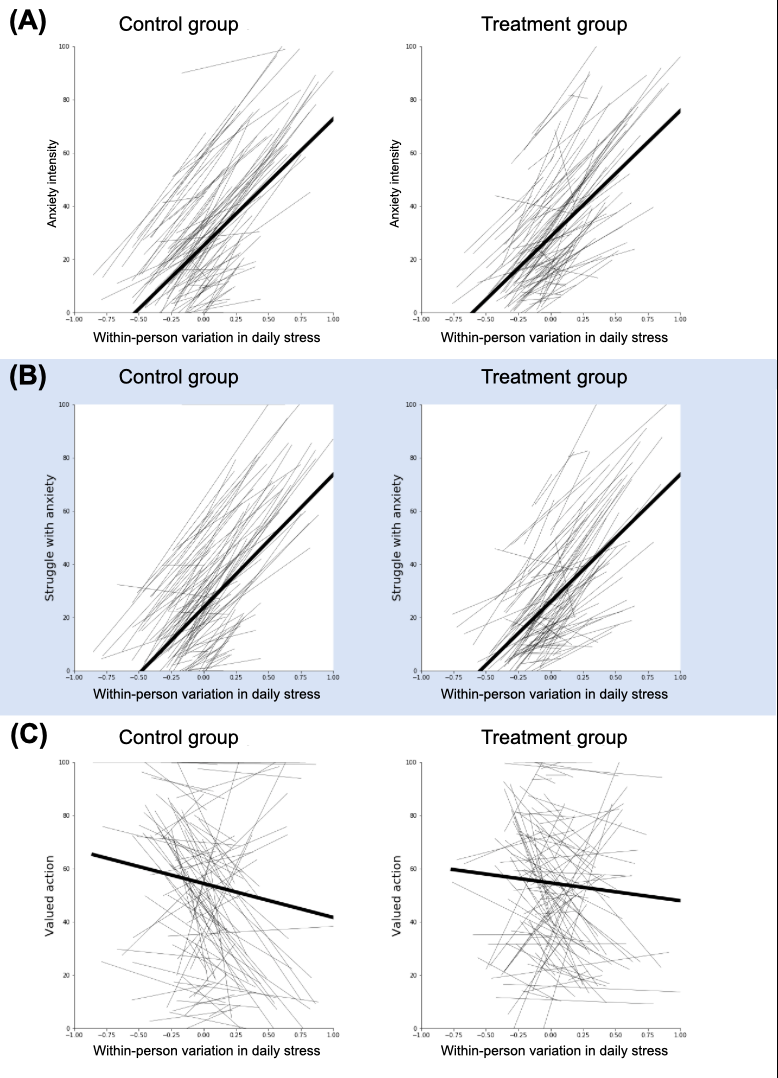


Figure S1. Spaghetti plots illustrating regression lines for individual participants (thin lines) and the average effect (thick lines). The panels present daily outcome measures in response to within-person variations in daily stress during the 7-days post-intervention period for the control group and the treatment group. Panel A) depicts the intensity of anxiety, Panel B) shows the struggle with anxiety, and Panel C) displays valued action.

Table S1. Results for the intensity of participants’ anxiety during the post-intervention phase. Each row corresponds to a predictor in a mixed linear model, with the intensity of anxiety as the dependent variable.

| Solution for Fixed Effects | | | | | | | |
| --- | --- | --- | --- | --- | --- | --- | --- |
| Effect | Estimate | Standard  Error | DF^a^ | t Value | P value^b^ | 95% Confidence Interval | |
|  |  |  |  |  |  | Lower | Upper |
| Intercept | 29.8648 | 3.6899 | 192 | 8.09 | <.0001 | 22.5869 | 37.1427 |
| Intensity pre^c^ | 0.1058 | 0.03896 | 197 | 2.72 | 0.0072 | 0.02896 | 0.1826 |
| Self-compassion pre | -2.5877 | 0.9689 | 197 | -2.67 | 0.0082 | -4.4985 | -0.6768 |
| Unrelated stressors | 0.6029 | 0.1383 | 197 | 4.36 | <.0001 | 0.3301 | 0.8757 |
| Condition | -0.482 | 1.2528 | 197 | -0.38 | 0.7009 | -2.9526 | 1.9886 |
| Between stress | 7.3362 | 0.6299 | 197 | 11.65 | <.0001 | 6.094 | 8.5785 |
| Condition*between stress | 0.3209 | 0.6866 | 197 | 0.47 | 0.6408 | -1.0332 | 1.675 |
| Within stress | 7.7719 | 0.3512 | 197 | 22.13 | <.0001 | 7.0792 | 8.4645 |
| Condition*within stress | -0.7745 | 0.493 | 197 | -1.57 | 0.0589 | -1.7466 | 0.1977 |
| Time^d^ | -0.07125 | 0.1393 | 197 | -0.51 | 0.6095 | -0.3459 | 0.2034 |
| Solution for Random Effects | | | | | | | |
| Cov Parm | Subject | Estimate | Standard  Error | Z Value | P value | 95% Confidence Interval | |
|  |  |  |  |  |  | Lower | Upper |
| UN(1,1) | ID | 56.3269 | 7.8223 | 7.2 | <.0001 | 43.6653 | 75.4533 |
| UN(2,1) | ID | 9.1001 | 1.9961 | 4.56 | <.0001 | 5.1879 | 13.0123 |
| UN(2,2) | ID | 4.1196 | 1.1115 | 3.71 | 0.0001 | 2.5843 | 7.5855 |
| SP(POW)^e^ | ID | 0.1158 | 0.04129 | 2.81 | 0.005 | 0.03491 | 0.1968 |
| Residual |  | 81.0821 | 4.1881 | 19.36 | <.0001 | 73.4613 | 89.9599 |

^a^ As advised by Bolger and Laurenceau (2013), we adopted a conservative approach in determining the degrees of freedom, utilizing the number of subjects (N = 197) rather than the total number of observations (N = x).

^b^ All *P*-values reported in the analysis are two-tailed, except for the interaction of condition*within stress, for which one-tailed p-values are used in accordance with the pre-registered directional hypotheses.

^c^ *Intensity pre* refers to the average intensity of participants’ anxiety during the 4-day pre intervention period.

^d^ As advised by Bolger and Laurenceau (2013), the variable time was rescaled such that 0 corresponds to the middle of the intervention period. A 1-unit difference in the variable time represents the passage of one day.

^e^ Although the autocorrelation in the data is statistically significant, it is relatively small in magnitude, with a value of 0.04. This indicates that there is a weak positive autocorrelation in the level-1 (within-subject) residuals.

Table S2. Results for the participants’ struggle with anxiety during the post- intervention phase. Each row corresponds to a predictor in a mixed linear model, with struggle with anxiety as the dependent variable.

| Solution for Fixed Effects | | | | | | | |
| --- | --- | --- | --- | --- | --- | --- | --- |
| Effect | Estimate | Standard  Error | DF^a^ | t Value | P value^b^ | 95% Confidence Interval | |
|  |  |  |  |  |  | Lower | Upper |
| Intercept | 23.8642 | 5.0701 | 191 | 4.71 | <.0001 | 13.8636 | 33.8647 |
| Struggle pre^c^ | 0.05061 | 0.03775 | 197 | 1.34 | 0.1816 | -0.02384 | 0.1251 |
| self-compassion pre | -2.6169 | 1.0785 | 197 | -2.43 | 0.0161 | -4.7437 | -0.49 |
| Believability of anxious thoughts pre | 1.2121 | 0.5926 | 197 | 2.05 | 0.0422 | 0.04337 | 2.3808 |
| unrelated stressors | 1.1274 | 0.1475 | 197 | 7.64 | <.0001 | 0.8365 | 1.4183 |
| condition | -1.6754 | 1.2161 | 197 | -1.38 | 0.1698 | -4.0736 | 0.7228 |
| between stress | 7.2582 | 0.5935 | 197 | 12.23 | <.0001 | 6.0877 | 8.4287 |
| condition*between stress | -0.5383 | 0.6683 | 197 | -0.81 | 0.4215 | -1.8562 | 0.7796 |
| within stress | 7.9335 | 0.3579 | 197 | 22.17 | <.0001 | 7.2277 | 8.6394 |
| condition*within stress | -1.4276 | 0.5019 | 197 | -2.84 | 0.00245 | -2.4174 | -0.4377 |
| Time^d^ | 0.1679 | 0.1506 | 197 | 1.12 | 0.2662 | -0.129 | 0.4648 |
| Solution for Random Effects | | | | | | | |
| Cov Parm | Subject | Estimate | Standard  Error | Z Value | P value | 95% Confidence Interval | |
|  |  |  |  |  |  | Lower | Upper |
| UN(1,1) | ID | 49.7465 | 7.4965 | 6.64 | <.0001 | 37.7944 | 68.4549 |
| UN(2,1) | ID | 8.9493 | 2.0686 | 4.33 | <.0001 | 4.8949 | 13.0037 |
| UN(2,2) | ID | 3.7447 | 1.1829 | 3.17 | 0.0008 | 2.193 | 7.8015 |
| SP(POW)^e^ | ID | 0.113 | 0.03999 | 2.82 | 0.0047 | 0.03457 | 0.1913 |
| Residual |  | 96.2213 | 5.0425 | 19.08 | <.0001 | 87.0554 | 106.92 |

^a^ As advised by Bolger and Laurenceau (2013), we adopted a conservative approach in determining the degrees of freedom, utilizing the number of subjects (N = 197) rather than the total number of observations (N = x).

^b^ All *P*-values reported in the analysis are two-tailed, except for the interaction of condition*within stress, for which one-tailed p-values are used in accordance with the pre-registered directional hypotheses.

^c^ *Struggle pre* refers to the average intensity of participants’ anxiety during the 4-day pre intervention period.

^d^ As advised by Bolger and Laurenceau (2013), the variable time was rescaled such that 0 corresponds to the middle of the intervention period. A 1-unit difference in the variable time represents the passage of one day.

^e^ Although the autocorrelation in the data is statistically significant, it is relatively small in magnitude, with a value of 0.04. This indicates that there is a weak positive autocorrelation in the level-1 (within-subject) residuals.

Table S3. Results for the participants’ enactment of their valued action during the post-intervention phase. Each row corresponds to a predictor in a mixed linear model, with valued intention enactment as the dependent variable.

| Solution for Fixed Effects | | | | | | | |
| --- | --- | --- | --- | --- | --- | --- | --- |
| Effect | Estimate | Standard  Error | DF^a^ | t Value | P value^b^ | 95% Confidence Interval | |
|  |  |  |  |  |  | Lower | Upper |
| Intercept | 13.4381 | 3.5836 | 192 | 3.75 | 0.0002 | 6.3698 | 20.5063 |
| Valued action pre^c^ | 0.7827 | 0.04246 | 197 | 18.44 | <.0001 | 0.699 | 0.8664 |
| Unrelated stressors | -0.5673 | 0.2661 | 197 | -2.13 | 0.0343 | -1.092 | -0.04251 |
| Condition | 4.4819 | 2.3236 | 197 | 1.93 | 0.0552 | -0.1003 | 9.0642 |
| Between stress | 1.2545 | 0.8768 | 197 | 1.43 | 0.1541 | -0.4746 | 2.9835 |
| Condition*between stress | -1.9358 | 1.2954 | 197 | -1.49 | 0.1367 | -4.4905 | 0.6188 |
| Within stress | -2.9483 | 0.8451 | 197 | -3.49 | 0.0006 | -4.6149 | -1.2816 |
| Condition*within stress | 1.6245 | 1.1929 | 197 | 1.36 | 0.0874 | -0.7279 | 3.9769 |
| Time^d^ | -0.08194 | 0.2845 | 197 | -0.29 | 0.7737 | -0.643 | 0.4792 |
| Solution for Random Effects | | | | | | | |
| Cov Parm | Subject | Estimate | Standard  Error | Z Value | P value | 95% Confidence Interval | |
|  |  |  |  |  |  | Lower | Upper |
| UN(1,1) | ID | 186.26 | 27.269 | 6.83 | <.0001 | 142.55 | 253.81 |
| UN(2,1) | ID | -29.0706 | 10.1419 | -2.87 | 0.0042 | -48.9483 | -9.1929 |
| UN(2,2) | ID | 34.7478 | 6.5496 | 5.31 | <.0001 | 24.787 | 52.231 |
| SP(POW)^e^ | ID | 0.1795 | 0.04453 | 4.03 | <.0001 | 0.09218 | 0.2667 |
| Residual |  | 310.01 | 16.93 | 18.31 | <.0001 | 279.33 | 346.07 |

^a^ As advised by Bolger and Laurenceau (2013), we adopted a conservative approach in determining the degrees of freedom, utilizing the number of subjects (N = 197) rather than the total number of observations (N = x).

^b^ All p-values reported in the analysis are two-tailed, except for the interaction of condition*within stress, for which one-tailed p-values are used in accordance with the pre-registered directional hypotheses.

^c^ *Valued action pre* refers to the average amount of valued action enacted by participants’s during the 4-days pre intervention period.

^d^ As advised by Bolger and Laurenceau (2013), the variable time was rescaled such that 0 corresponds to the middle of the intervention period. A 1-unit difference in the variable time represents the passage of one day.

^e^ Although the autocorrelation in the data is statistically significant, it is relatively small in magnitude, with a value of 0.04. This indicates that there is a weak positive autocorrelation in the level-1 (within-subject) residuals.
